# RNA-Binding Proteins: The Key Modulator in Stress Granule Formation and Abiotic Stress Response

**DOI:** 10.3389/fpls.2022.882596

**Published:** 2022-06-15

**Authors:** Yanyan Yan, Jianghuang Gan, Yilin Tao, Thomas W. Okita, Li Tian

**Affiliations:** ^1^Key Laboratory of Quality and Safety Control for Subtropical Fruit and Vegetable (Ministry of Agriculture and Rural Affairs), Collaborative Innovation Center for Efficient and Green Production of Agriculture in Mountainous Areas of Zhejiang Province, College of Horticulture Science, Zhejiang A&F University, Hangzhou, China; ^2^Institute of Biological Chemistry, Washington State University, Pullman, WA, United States

**Keywords:** RNA-binding proteins, stress granules (SGs), RNA metabolism, stress response, post-transcriptional gene regulation

## Abstract

To cope with abiotic environmental stress, plants rapidly change their gene expression transcriptionally and post-transcriptionally, the latter by translational suppression of selected proteins and the assembly of cytoplasmic stress granules (SGs) that sequester mRNA transcripts. RNA-binding proteins (RBPs) are the major players in these post-transcriptional processes, which control RNA processing in the nucleus, their export from the nucleus, and overall RNA metabolism in the cytoplasm. Because of their diverse modular domain structures, various RBP types dynamically co-assemble with their targeted RNAs and interacting proteins to form SGs, a process that finely regulates stress-responsive gene expression. This review summarizes recent findings on the involvement of RBPs in adapting plants to various abiotic stresses *via* modulation of specific gene expression events and SG formation. The relationship of these processes with the stress hormone abscisic acid (ABA) is discussed.

## Introduction

A major molecular response by plants to environmental stress is the rapid reprogramming of gene expression, which impacts the proteome and cellular metabolism to achieve an equilibrium between growth, development and survival (Glisovic et al., 2008; Zhang et al., 2020). Growing evidence from global transcript profiling studies and the discovery of RNA granules, especially stress granules (SGs), have brought about the importance of post-transcriptional gene regulation into sharper focus during the plant’s adaptation to stress (Buchan et al., 2013; Bach-Pages et al., 2020). Post-transcriptional gene regulation largely relies on RNA-binding proteins (RBPs). RBPs recognize and bind to specific target RNAs to modulate the activity and fate of RNA transcripts (Marondedze, 2020). The association of RBPs with RNAs may begin as early as transcription in the nucleus and persist until RNA degradation in the cytoplasm. The spatio-temporal binding of RBPs with target RNAs occurs at various stages of RNA metabolism to dynamically regulate specific processes such as splicing, processing, transport, localization and decay. Some RBPs possess DNA-melting or RNase activities and thus function as RNA chaperones to facilitate or suppress RNAs from forming functional or deleterious secondary or tertiary conformational structures. The properly structured RNAs, together with specific RNA sequences, may further act as a binding signal to recruit other RBPs, which collectively mediates the precise control of RNA processing, RNA transport, and gene expression. With such critical roles by RBPs, plants can modulate the abundance of individual RNAs, and thus finely tune translational control of protein expression to rapidly respond and adapt to plant stress as described in several reviews (Kwak et al., 2016; Marondedze, 2020; Muthusamy et al., 2021). To obtain a more precise view of post-transcriptional gene regulation during plant stress, we review here the recent advances on the functions of RBPs in modulating specific gene expression and the formation of stress granules during plant adaptation to abiotic stress induced by salt, drought, heat and cold as well as that mediated by oxidation, hypoxia and flooding. Lastly, the interplay between RBPs and stress hormone abscisic acid (ABA) will be discussed.

## Plant RNA-Binding Proteins and Abiotic Stress Response

RNA-binding proteins are highly conserved proteins in eukaryotes and diverse in their ability to interact with RNAs to regulate post-transcriptional events. RBPs are typically characterized by the presence of one or more RNA binding domains (RBDs). These include the RNA recognition motif (RRM), K homology (KH) domain (Lorković and Brarta, 2002), zinc finger domain (mainly C-×8-C-×5-C-×3-H type) (Kim Y.O. et al., 2007; Kim et al., 2010b), double-stranded RNA binding domain (DS-RBD) (Masliah et al., 2013), cold shock domain (CSD) (Sasaki and Imai, 2011), Pumilio/FBF (PUF) domain (Tam et al., 2010), and the DEAD/DEAH boxes (Asp-Glu-Ala-Asp/His motif) highly conserved in RNA helicases (Owttrim, 2006). Among these domains, the RNA recognition motif (RRM) is the most abundant domain/motif among RNA-binding proteins (Nakaminami et al., 2012) as exemplified in the *Arabidopsis* genome where 197 out of 800 RBPs contain RRM motifs (Lorković and Brarta, 2002). The predominant role of RBDs involves RNA recognition and protein-protein interactions, leading to the formation of heterogeneous ribonucleoprotein (RNP) complexes (Maris et al., 2005). In addition to RNA binding domains, most RBPs contain auxiliary domains or motifs at the N- or C-terminal region, which many serve as protein interacting regions. These include the glycine-rich region, arginine-rich domain, arginine-glycine (RGG), arginine/aspartic acid (RD)-repeats, and serine-arginine (SR) repeats (Nagai et al., 1995; Albà and Pagès, 1998). According to their structural and binding specificity, RNA-binding proteins are also classified as glycine-rich RNA-binding proteins (GR-RBP, also named as GRP), zinc finger glycine-rich proteins (RZ), cold shock domain proteins (CSDP), DEAD-box RNA helicases (RH), chloroplast RNA splicing and ribosome maturation domain proteins (CRM), S1 domain-containing proteins (SDP), and pentatricopeptide repeat proteins (PPR) (Lee and Kang, 2020). The diverse structures of RBPs suggest a variety of functions among the various RBP families (Lee and Kang, 2016). In this review, we focus on the functional roles of the abovementioned typical RBPs, including GR-RBPs, RZs, CSDPs, RHs, SRs, PPRs, TZFs, SDPs, and CRMs as well as several known classic proteins, including Tudor-SN and RBPs containing RRM, RBD, and RGG RNA binding domains.

**FIGURE 1:**
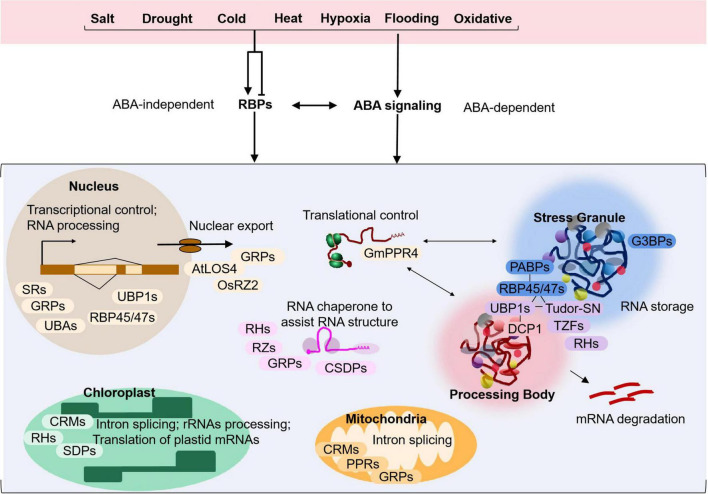
Model depicting the regulatory functions of the typical RNA-binding proteins in plant adaptation to abiotic stress. Environmental stress caused by salt, drought, cold, heat, hypoxia, flooding or oxidative conditions may induce or repress the expression of relevant RBPs. During the response, RBPs may act in a ABA-dependent or independent pathway to regulate gene expression and play various roles in RNA metabolism including RNA processing and alternative splicing in the nucleus, nuclear export of mRNAs, mRNA degradation *via* processing bodies, mRNA storage in stress granules, and translational control in the cytoplasm. Some RBPs may also function as RNA chaperones to assist RNA folding and structure remodeling. Nuclear-encoded RBPs may also be targeted to chloroplasts or mitochondria and participate in intron splicing, rRNAs processing and/or translation of plastid mRNAs, processes critical for organellar biogenesis and function during plant adaptation to stress. Examples of RBPs that are involved in each cellular process are shown in the model. More detailed information can be found in Table 1 and in the main text.

The application of high-resolution multi-omics techniques have identified an increasing number of RBPs as crucial factors in regulating plant stress response. In a recent label-free mass spectrometry study in *Arabidopsis* (Marondedze et al., 2019), 567 proteins with potential RNA-binding activity are highly enriched in drought stress-induced samples, suggesting that plants utilize RBPs as a pervasive regulatory response during plant stress. As shown in Table 1 and Figure 1, RBPs are involved in abiotic stress conditions under salt, drought, cold, heat, hypoxia, flooding and oxidative stress, and play a comprehensive function in stress responding processes.

**TABLE 1:** Plant RBPs involved in abiotic stress response and SG formation.

RBP types	Domain(s)^1^	RBPs^2^	Location^3^	Abiotic Stress (± /s)^4^	ABA^5^	SGs^6^	Functions and description in stress	References
GR-RBPs	GR, RRM	AtGRP1	Nc, Cy	Salt(+)	−	−	−	Wang et al., 2012
		AtGRP2	Nc, Cy	Drought(−); Cold(+)	−	−	−	Flores and Sachetto-Martins, 2007; Kim Y.O. et al., 2007; Yang et al., 2014; Ciuzan et al., 2015
		AtGRP4	Nc, Cy	Salt(−); Drought(−); Cold(+);Heat(−); Oxidative(−)	−	−	Function as RNA chaperone to assist folding of RNA structure	Kwak et al., 2005, 2011; Kim Y.O. et al., 2007
		AtGRP7	Nc, Cy	Drought(+); Cold(+); Heat(+);Oxidative(+)	−	−	Nuclear export of mRNA transcripts; Regulate stomatal opening and closing in the guard cells under abiotic stresses	Cao et al., 2006; Kim J.S. et al., 2007; Kim et al., 2008, 2010a; Schmidt et al., 2010; Kwak et al., 2011;
		AtGRP8	Nc, Cy	Cold(+); Oxidative(−)	−	−	−	Schmidt et al., 2010
		AtRBDG2,4	Nc, Cy	Heat(+)	−	√(+)	Participate in SG formation	Zhu et al., 2022
		OsGRP1,4,6	Nc, Cy	Cold(+)	−	−	Function as RNA chaperone	Kim et al., 2010a
		OsGRP3	Nc, Cy	Drought(+)	√(+)	−	Function as RNA chaperone	Shim et al., 2021
		NtGRP1	Nc, Cy	Salt(+); Drought(+); Cold(+); Heat(+); Flooding(+)	√(+)	−	−	Lee et al., 2009; Khan et al., 2013
		NtGRP1a, 1b,2,3	Nc, Cy	Salt(−); Drought(+); Cold(+); Heat(+); Flooding(+)	×	−	Function as a negative modulator of gene expression by binding to DNA or RNA in bulk	Molina et al., 1997; Nomata et al., 2004; Shinozuka et al., 2006; Khan et al., 2013; Long et al., 2013; Kwak et al., 2016; Wang et al., 2018; Huang et al., 2019
		EsCOR20	−	Cold(+)	−	−	Hybridize to RNAs	Horvath and Olson, 1998
		LbGRP1	Nc, Cy	Salt(+)	−	−	Restrict the entry of Na^+^ reduce potassium loss under salt stress	Wang et al., 2012
		LpGRP1	Nc, Cy	Cold(+)	√(+)	−	Involved in pre-mRNA processing	Shinozuka et al., 2006
		MsGRP	Cm, Cw	Salt(+); Drought(+)	√(+)	−	−	Long et al., 2013
		NgRBP	Nc, Cy	−	√(+)	−	−	Huang et al., 2019
		CsGR-RBP3	Mt	Drought(+); Cold(+)	√(−)	−	Modulated antioxidant enzymes	Wang et al., 2018
		CsGRP7-a	Nc, Cy	Salt(−); Cold(+)	−	−	−	Kwak et al., 2016
		HvGRP2, 3	Nc, Cy	Cold(+)	−	−	−	Molina et al., 1997
		PpGRP3	Mt	Cold(+)	−	−	Associate with post-transcriptional processing of mitochondrial RNA	Nomata et al., 2004
RZ	ZF, RRM, GR	AtRZ-1a AtRZ-1b	Nc, Cy	Cold(+); Salt(−); Drought(−) Cold(+)	√(−) −	−	Modulate the expression of genes involved in reactive oxygen species homeostasis and functions Function as RNA chaperone	Kim J.S. et al., 2007; Kim et al., 2010b
		OsRZ2	Nc, Ch	Cold(+)	−	−	Function as RNA chaperone to regulate mRNA export from the nucleus	Kim et al., 2010b
		BrRZ1, 2, 3	Nc	Salt(+); Drought(+); Cold(+)	√(+)	−	Function as RNA chaperone	Park et al., 2017
		TaRZ2, 3	Nc	Salt(+); Drought(−); Cold(+)	−	−	−	Xu et al., 2014
CSDP	CSD, ZF, GR	AtCSDP 1	−	Drought(−); Cold(−)	−	−	Function as RNA chaperone; Prefer binding to poly(G) and poly(A) sequence	Park et al., 2009
		AtCSDP 2	−	Salt(+)	−	−	Strong binding to poly(U)	Park et al., 2009
		AtCSDP3	−	Drought(−); Cold(+)	−	−	−	Park et al., 2009
		OsCSDP1,2	−	Cold(+)	−	−	−	Chaikam and Karlson, 2008
		BrCSDP3	Nc, Ch	Salt(+); Drought(+); Cold(+)	√(+)	−	−	Choi et al., 2015
RH	DEAD-box	OsRH58	Ch	Salt(+); Drought(+); Cold(−); Heat(+)	√(−)	−	Modulate the expressions of stress responsive genes	Nawaz and Kang, 2019
		AtRH50	Ch	Cold(+)	−	−	associated with plastid gene expression	Paieri et al., 2018
		AtRH9,25	−	Salt(−); Drought(−)	−	−	−	Kim et al., 2008
		AtRH3	Ch	Salt(+); Drought(+); Cold(+)	−	−	Function as RNA chaperone; Involve in intron splicing, ribosome biogenesis	Gu et al., 2014
		OsTCD33	Ch	Cold(+)	−	−	Modulate the expression of cold responsive gene	Wang et al., 2020
		BrRH22	Ch	Salt(+);Drought(+); Cold(+); Heat(+); UV(−)	√(+)	−	Function as RNA chaperone; affect translation of chloroplast transcripts.	Nawaz et al., 2018
		AtRH17	−	Salt(+)	−	−	−	Nguyen et al., 2018
		AtRH7	−	Cold(+)	−	−	Participate in pre-rRNA processing	Huang et al., 2016
		AtSTRS1, 2	Nc	Salt(−); Heat(−);Osmotic (−)	√(−)	−	Attenuate the expression of stress-responsive transcriptional activators	Kant et al., 2007
		AtLOS4	Nc	Heat(+)	√(−)	−	Regulate RNA export	Gong et al., 2005
		AtDHH1/DDX6	Cy	Hypoxia(+)	−	√(+)	Physically associate with both PBs and SGs; mediate translation inhibition and mRNA degradation	Chantarachot et al., 2020
		SlDEAD31	−	Salt(+); Drought(+)	−	−	Modulating the expressions of stress responsive genes	Zhu et al., 2015
		OsTCD10	Ch	Cold(+)	−	−	Recognizing single stranded RNA sequences	Wu et al., 2016
SR	RRM, RS	AtSR45a-1a, 1b	−	Salt(−)	−	−	Participate in alternative splicing and mRNA maturation	Li et al., 2021
		BrSR45a	−	Drought(+)	−	−	Participate in alternative splicing of drought-stress response genes	Muthusamy et al., 2020
PPR	PPR	AtSOAR1	Nc, Cy	Salt(+); Drought(+); Cold(+)	√(−)	−	Recognize single-stranded RNA targets	Jiang et al., 2015
		AtPGN (PPR)	Mt	Salt(+)	√(−)	−	Recognize single-stranded RNA targets	Laluk et al., 2011
		GmPPR4	−	Drought(+)	−	−	Function in RNA splicing, stabilization, and translational activation	Su et al., 2019
		AtPPR96,40	−	Salt(+)	−	−	−	Liu et al., 2016
TZF	TZF	AtTZF1	Nc, Cy	Salt(+); Heat(+); Hypoxia(−)	√(+)	√	Associate with both SGs and PBs; AtTZF1shuttle between nucleus and cytoplasmic PBs under normal condition, but predominantly target to SG-like foci during heat stress	Pomeranz M. et al., 2010, Pomeranz M.C. et al., 2010; Lin et al., 2011; Bogamuwa and Jang, 2014; Bogamuwa and Jang, 2016; Han et al., 2021
		AtTZF2,3	Nc, Cy	Salt(+); Heat(+); Hypoxia(−)	−	√		
		AtTZF4,7,8	Cy	Salt(+); Hypoxia(−)	−	√		
		AtTZF5	Cy	Heat(−)	−	√		
		AtTZF6	Cy	Salt(+)	−	√		
		AtTZF10,11	Cy	Salt(+); Hypoxia(+)	−	√		
		OsTZF1	−	Salt(+); Drought(+)	√(+)	√	Associate with both SGs and PBs; regulate the expression of genes related to stress, reactive oxygen species homeostasis, and metal homeostasis.	Jan et al., 2013
G3BP	NTF, RRM, RGG	AtG3BP1	Cy	Cold(+); Heat(+); Oxidative(−); High Light(+)	×	√	All AtG3BPs interact with each other, and interact with AtUBP-24 in SG-like granules.	Zimmermann et al., 2004; Abulfaraj et al., 2018, 2021; Reuper et al., 2021
		AtG3BP2	Cy	Cold(+); Heat(−); Oxidative(−)	×	√		
		AtG3BP3	Cy	Cold(+); High Light(+)	√(+)	√		
		AtG3BP4	Cy	Heat(+); Oxidative(−)	×	√		
		AtG3BP5	Cy	Cold(+)	√(+)	v		
		AtG3BP6	Cy, Nc	Cold(−); Heat(+); Oxidative(−)	×	√		
		AtG3BP7	Cy	Cold(+); Oxidative(−); High Light(+)	×	√		
		AtG3BP8		Oxidative(−)	×	√		
SDP	SDP	AtSRRP1	Ch		√(+)	−	Function as RNA chaperone; splicing of trnL intron and processing of 5S rRNA in chloroplast	Gu et al., 2015
		AtRPS5	Ch	Cold(+)	−	−	Participate in processing of *16S rRNA* in chloroplast	Zhang et al., 2016
		AtSDP	Ch	Salt(+);Heat(+); UV(+); Cold(+); Drought(×)	×	−	Participate in processing of *16S, 23S, 4.5S*, and *5S rRNAs* in chloroplast	Dinh et al., 2019
CRM	CRM	AtCFM4	Ch	Salt(+); Cold(+)	√(+)	−	Function as RNA chaperone; Participate in processing of *16S* and *23S rRNA* processing in chloroplast;	Lee et al., 2014
		AtCFM9	Mt	Salt(+);Drought (+)	√(+)	−	Participate in splicing of mitochondrial genes	Lee et al., 2019
Others	RRM	AtCBP20	−	Drought(−)	√(−)	−	Interact with CBP80	Papp et al., 2004
		AlSRG1	−	Salt(+);Osmotic (+)	−	−	Regulate the expression of tROS-scavenging genes and stress-responsive transcription factors	Saad et al., 2018
		OsDEG10	−	Salt(+); Cold(+); High Light(+)	√(+)	−	−	Park et al., 2009
		OsRBD1	Nc	Salt(+); Drought(+)	−	−	Interacts with OsSRO1a to regulate stress and hormonal response	Sharma et al., 2016
		(At)cpRNP29; AtCSP41B	Ch		√(−)	−	Participate in chloroplast RNA metabolism	Raab et al., 2006
		AtUBP1a	Nc, Cy	Hypoxia(s)	−	√	Modulate SG formation; associate with selective mRNAs and protect stress-related mRNAs from degradation during heat stress; Links SGs with PBs possibly *via* interaction with PB marker DCP1	Sorenson and Bailey-Serres, 2014
		AtUBP1b	Nc, Cy	Salt(s); Heat(s)	√(s)	√	−	Weber et al., 2008
		AtUBP1c	Nc, Cy	Hypoxia(s)	√(+)	√	−	Sorenson and Bailey-Serres, 2014; Nguyen et al., 2016, 2017
		AtUBA2a, AtUBA1a	Nc	−	√(+)	√	Reorganize in the nuclear speckles under ABA and stress; Interact with UBP1; regulate pre-mRNA splicing;	Lambermon et al., 2002; Riera et al., 2006; Bove et al., 2008
		AtRBP45,47	Nc, Cy	Heat(s)	−	√	Interacts with poly(A)^+^ RNA and regulates pre-mRNA maturation in nucleus; key component of SGs; RBP47 interacts with UBP1, PABPs and 2′,3′-cAMP during SG formation, and recruits angustifolia protein (AN) to assemble SGs under stress conditions.	Lorković et al., 2000; Weber et al., 2008; Yan et al., 2014; Gutierrez-Beltran et al., 2015; Hemal and Martin, 2017; Kosmacz et al., 2018, 2019
	PUF	AtAPUM5	Nc	Salt(+); Drought(+)	√(+)	−	Regulates gene expression through direct binding to 3′UTRs	Huh and Paek, 2014
	Tudor, SN	AtTudor-SN	Cy	Salt(+); Heat(s)	√(+)	√	Component of SGs; Co-localize with RBP47 in SGs; function as docking platform for SG formation; Associate with both SGs and PBs.	Dit Frey et al., 2010; Yan et al., 2014; Gutierrez-Beltran et al., 2016, 2021
	ZF	AtSRP1	Nc	Salt(−); Cold(−)	√(−)	−	Bindsto *ABI2* 3′UTR and regulate its expression; Regulates the expression of ABA signaling-related genes.	Xu et al., 2017
	MIF4G	AtABH1	Nc	Drought(−)	√(−)	−	Modulate of ABA-related stomatal closing and cytosolic calcium level	Hugouvieux et al., 2001
	LSM	AtSAD1	−	Drought(−)	√(−)	−	Regulation of ABA signaling genes	Xiong et al., 2001
	dsRBD	AtHYL1	−	Drought(−)	√(−)	−	−	Lu and Fedoroff, 2000
	RGG	AtRGGA	Cy	Drought(+)	√(+)	−	−	Ambrosone et al., 2010
	HAT, TPR, PRP1, UBQ	AtSTA1	Nc	Cold(+)	√(+)	−	Participate in pre-mRNA splicing and mRNA turnover	Lee et al., 2006
	PABC	AtPABP2,8	Cy	Heat(s); Hypoxia(s)	−	√	Localize to SGs and show similar kinetics as eIF4E in SGs; Interact with RBP47.	Weber et al., 2008; Sorenson and Bailey-Serres, 2014

*^1^Description of domains: RRM, recognition RNA motif; GR, glycine-rich; CSD, cold shock domain; PPR, pentatricopeptide repeat; PUF, pumilio/fem-3 binding factors; dsRBD, double-stranded RNA (dsRNA)-binding domain; TPR, tetratricopeptide repeat (TPR); TZF, tandem zinc-finger motifs; SDP, S1 domain-containing protein; CRM, chloroplast RNA splicing and ribosome maturation; HAT, Half-A-TPR (HAT); UBQ, ubiquitin; ZF, zinc-finger; SN, staphylococcal nuclease-like domain; LSM, Sm-like; RGG, arginine-glycine rich; CRM, Chloroplast RNA splicing and ribosome maturation; SDP, S1 domain-containing; PABC, poly(A)-binding protein C-terminal domain.*

*^2^Description of species: At, Arabidopsis thaliana; Os, Oryza sativa; Nt, Nicotiana tabacum; Cs, Cucumis sativus; Br, Brassica napus; Sl, Solanum lycopersicum; Cs, Cucumis sativus; Ta, Triticum aestivum; Gm, Glycine max; Lb, Limonium bicolor; Lp, Lolium perenne; Ms, Medicago sativa; Hv, Hordeum vulgare; Ng, Nicotiana glutinosa; Pp, Physcomitrella patens; Es, Euphorbia esula.*

*^3^Description of Location: Nc, nucleus; Cy, cytoplasm; Cw, cell wall; Cm, cell membrane; Ch, chloroplast; Mt, mitochondria.*

*^4^Description of response to abiotic stress: +, positive regulation; −, negative regulation; s, stress granule related.*

*^5^Relationship between RBP and ABA: √(+), induced by ABA; √(−), repressed by ABA; ×, no response to ABA.*

*^6^Interaction with SGs: √means the protein localizes in SGs or participates in SG formation.*

*−, unknown or not detected.*

A well-known abiotic stress associated RBP is GR-RBP. GR-RBPs belong to group IV of glycine-rich proteins (GRPs) superfamily, whose members possess a glycine-rich region at the C-terminal and RRM at the N-terminal end (Mangeon et al., 2010; Ortega-Amaro et al., 2014). Multiple lines of evidence suggest GR-RBPs are strongly associated with temperature stress. In *Arabidopsis*, AtGRP2 and AtGRP7 promote seed germination and seedling growth at low temperature (Cao et al., 2006; Kim J.S. et al., 2007; Schmidt et al., 2010; Kwak et al., 2011). Interestingly, AtGRP7 increases the viability of *Escherichia coli* under cold shock (Kim et al., 2010a). In rice, OsGRP1, OsGRP4 and OsGRP6 accelerate seed germination and seedling growth under cold stress and can rescue *Arabidopsis grp7* knockout plants under cold conditions (Kim et al., 2010a). The expression of *LpGRP1* mRNAs was significantly increased in root, crown and leaf tissues of a perennial ryegrass under freezing treatment (Shinozuka et al., 2006). A cucumber mitochondrial-located CsGR-RBP3, when down-regulated, significantly aggravated chilling injury while its overexpression conferred *Arabidopsis* a high survival rate under low temperature (Wang et al., 2018). In addition to cold stress, the *Arabidopsis* AtGRP2, AtGRP4, and AtGRP7 (Kwak et al., 2005; Cao et al., 2006) and LbGRP1 from *Limonium bicolor* (Wang et al., 2012) were also reported to be involved in salt and osmotic stresses.

Although the involvement of GR-RBP in plant stress response can be traced back to the discovery of a glycine rich protein from maize induced by drought in 1988 (Gómez et al., 1988) and *AtGRP5* (previously named M16) response to flooding stress in 1995 (Sachetto-Martins et al., 1995), the functional role of GR-RBPs under these stress conditions is still unclear. In the case of AtGRP7, transcriptome analysis showed that overexpression of *AtGRP7* alters the expression of stress-related plant defensins and pathogenesis-related proteins (Streitner et al., 2010). Experimental evidence suggests that AtGRP7 has DNA melting activity and enhance RNase activity (Kim J.S. et al., 2007), which may prevent the formation of adverse RNA secondary structures likely stabilized at low temperatures, thus enabling them to be efficiently processed, exported, or translated (Sahi et al., 2007; Lorković, 2009). The role of AtGRP7 as a shuttle protein to promote mRNA export from the nucleus to the cytoplasm may further contribute to post-transcriptional regulation under cold stress (Kim et al., 2008). Those studies suggest that GR-RBPs may function as RNA chaperone under stress response (Kim et al., 2010b; Xu et al., 2014). The modular structure of GRPs likely directly contributes to these functions. While the N-terminal RRM is responsible for the nucleic acid-binding and RNA chaperone activities of AtGRP7, this region also confers higher growth-stimulating activity than its C-terminal region in *E. coli* under cold stress (Kim J.S. et al., 2007), suggesting the crucial role of the N-terminal region in cold response.

Zinc finger glycine-rich proteins (RZs) are another type of group IV GRPs, which contain a CCHC-type zinc finger domain instead of RRM. The *Arabidopsis* genome contains three RZ genes; AtRZ-1a, AtRZ-1b, and AtRZ1-c (Lorković and Brarta, 2002; Kim Y.O. et al., 2007). Similar to GR-RBPs, loss of *AtRZ-1* function affects seed germination and seedling growth at low temperature, while its overexpression enhances freezing resistance in *Arabidopsis* (Kim et al., 2005). Different from the case under cold stress, however, AtRZ-1a plays a negative role under salt or dehydration stress conditions as its overexpression retards germination and seedling growth under these stress conditions (Kim Y.O. et al., 2007). Proteomic analysis of a overexpression line in comparison with wild-type showed that AtRZ-1a modulates the expression of several germination-responsive genes (Kim Y.O. et al., 2007) including those related to reactive oxygen species homeostasis that are closely connected with the plant abiotic stress response. In rice, RZs may also function as RNA chaperone under cold stress (Kim et al., 2010a). While expression of the three rice RZ genes remain unchanged under salt and dehydration stress, their expression is up-regulated under cold stress. Interestingly, of the three rice RZs, only OsRZ2 could rescue cold-sensitive *Arabidopsis grp7* knockout plants from cold and freezing damage. Biochemical and cellular studies show that OsRZ2 possesses DNA-melting activity and transcription anti-termination activity, and complements the defect in mRNA export from the nucleus to the cytoplasm in *grp7* mutant. These findings suggest that the function of OsRZ2 as a RNA chaperone may contribute to cold resistance.

Another representative cold responding RBPs belong to the cold shock domain proteins (CSDPs) family. Similar to the cold shock protein (CSP) in prokaryotes, the plant CSDPs contain a cold shock domain (CSD). The CSD is highly conserved nucleic acid binding domain with the dual capability in binding DNA and single-stranded RNA (Hunger et al., 2006). In addition to the CSD domain, plant CSDPs usually possess additional glycine-rich regions interspersed with multiple CCHC-type zinc finger at the C-terminus (Chaikam and Karlson, 2008). Although the properties of bacterial CSPs have been well established, the functions of plant CSDPs have yet to be fully resolved. Recent studies suggest that some CSDPs such as the *Arabidopsis* AtCSDP1 and AtCSDP3 (Kim et al., 2009), the cabbage BrCSDP3 (Choi et al., 2015), and the wheat and rice CSDPs perform as RNA chaperones (Kim J.S. et al., 2007) enabling RNAs to attain a functionally active state *in vivo*. This can be accomplished by promoting or preventing RNA-RNA interactions and eliminating non-functional conformational structures (Rajkowitsch et al., 2007), which can impact the molecular fate of RNA and thus help plants prevent or overcome cellular stress damage under adverse conditions. In *Arabidopsis*, expression of AtCSDP1 and AtCSDP3 is induced by cold stress (Kim et al., 2009). Mutant *AtCSDP3* display increased plant sensitivity to low temperature, while overexpression of *AtCSDP3* enhances plant tolerance to cold stress (Kim et al., 2009). AtCSDP2 possesses nucleic acid melting activity (Sasaki et al., 2007) and is able to complement the cold sensitive *E. coli* BX04, a quadruple deletion mutant of cold shock domain proteins. In rice, OsCSDP1 and OsCSDP2 play a similar role and have the ability to bind nucleic acid as well (Chaikam and Karlson, 2008). Notably, different from GRPs that prefer to bind poly(U) sequence, AtCSDP1 binds preferentially to single-stranded DNA and G-rich RNAs (Kim J.S. et al., 2007).

C-terminal CCHC-type zinc fingers in CSDPs are reported to be essential for nucleic acid-binding and RNA chaperone activity. A CSDP gene lacking the C-terminal zinc fingers is unable to fully recover growth of bacterial BX04 cells. Conversely, the C-terminal region of AtCSDP comprising seven zinc fingers has a stronger growth-stimulating activity than the N-terminal region under cold stress (Kim J.S. et al., 2007).

Another prominent candidate for RNA chaperone activity under stress condition is the DEAD-box RNA helicases. RNA helicases (RHs) are ATP-dependent enzymes, which unwind double-strand RNAs and participate in multiple steps of RNA metabolism (Cruz et al., 1999; Tanner and Linder, 2001; Lorsch, 2002). As its name implies, DEAD-box RNA helicases usually contain the amino acids Asp-Glu-Ala-Asp (DEAD) box, which comprise the largest subgroup of RNA helicases. Several DEAD-box RNA helicases are found to participate under various stress conditions. The nucleus-located DEAD-box RNA helicase from *Arabidopsis*, previously named LOS4 (low expression of osmotically responsive genes 4) (Gong et al., 2005), is highly enriched at the nuclear rim. Mutant LOS4 have reduced content of poly(A)^+^ RNAs at high temperature, suggesting that LOS4 may function as essential factor to regulate RNA export under heat stress. The rice OsRH42 is tightly coupled to temperature stress with a specific location in nuclear speckles to support pre-mRNA splicing at low temperature (Lu et al., 2019). DH1 from the *halophyte Apocynum venetum*, a typical helicase that unwinds DNA and RNA, is involved in the response of plants to salinity stress (Liu et al., 2008). Cold-induced rice TCD33 with the DEAD-box RNA helicase domain is believed to be involved in chloroplast ribosome assembly and has been shown to affect chloroplast biogenesis under cold stress (Wang et al., 2020). In addition, chloroplast localized AtRH3, OsRH58 and BrRH22 contribute to structural rearrangement of target mRNA through their RNA chaperone activity, thus influencing chloroplast mRNA translation for subsequent efficient translation control under stress (Gu et al., 2014; Nawaz et al., 2018; Nawaz and Kang, 2019). The ectopic expression of the rice *OsRH58* or cabbage *BrRH22* confers increased tolerance of Arabidopsis to cold stress presumably by stimulating the translation of chloroplast mRNAs such as *POR*, *RBCL*, *CLPB3*, *PSBA*, and *PETA* transcripts (Nawaz et al., 2018; Nawaz and Kang, 2019).

The association of chloroplast-located RHs with stress response readily supports the involvement of organelle-located RBPs in acclimating plants to environmental stress. Other organellar RBPs possessing S1 RNA-binding domain (SDP), chloroplast RNA splicing and ribosome maturation (CRM) domain, or pentatricopeptide repeats (PPR) are also reported to function as RNA chaperones in assisting the correct folding of target RNA structure during plant growth and development, as well as under abiotic stress. S1 domain containing-protein (SDP), first identified in the *E. coli* ribosomal protein S1 (RPS1), has the ability to bind RNA during RNA degradation and protein synthesis (Subramanian, 1983; Francesco et al., 2011). Nuclear-coded chloroplast SDPs play crucial roles in chloroplast biogenesis and photosynthesis (Dinh et al., 2019; Lee and Kang, 2020). The chloroplast *16S*, *23S*, *4.5S rRNAs* are severely damaged in *sdp* mutant lines, which are unable to survive on sucrose deficient media due to defective photosynthesis (Han et al., 2015). The effects on rRNA processing in chloroplasts contributes to their positive function during UV, salt, heat or freezing stress tolerance (Dinh et al., 2019).

Chloroplast RNA splicing and ribosome maturation (CRM) proteins, first described in Archaea and eubacteria (Asakura and Barkan, 2007; Jacobs and Kück, 2011), contain a highly conserved GxxG sequence in the loop of the CRM domain. An *Arabidopsis* mitochondrial CRM Protein 9 (AtCFM9), which mediates the splicing of many intron-containing genes, is required for normal mitochondrial function. It plays an active role in seed germination and seedling growth under normal conditions as well as during ABA treatment, high salinity, or dehydration stress (Lee et al., 2019). Likewise, the chloroplast-localized CFM4 protein is also essential for normal seed germination and seedling growth. Unlike the mitochondria-localized protein, which is required for intron splicing, CFM4 is required for normal processing of chloroplast *16S* and *4.5S* ribosomal genes (Lee et al., 2014).

Pentatricopeptide repeat (PPR) proteins usually fold into a pair of antiparallel α helices, ranging from 2 to 30 tracts, and contribute to organellar RNA metabolism (Small and Peeters, 2000; Small et al., 2020). Chloroplast-localized PPR proteins, WSLs (Tan et al., 2014; Liu et al., 2018), OsV4 (Gong et al., 2014), and TCD10 (Wu et al., 2016) from rice, are involved in cold stress by affecting the splicing of chloroplast RNA transcripts *rpl2*, *rpl21*, and *rps12* as well as *16s rRNA*. Overexpression of mitochondria-localized *PPR40* in *Arabidopsis* promotes seed germination and seedling growth under treatment of high salinity or ABA by reducing reactive oxygen species (ROS) damage in the mitochondria (Zsigmond et al., 2008). Loss-of-function of the PGN (Pentatricopeptide Repeat Protein for Germination on NaCl) gene in *Arabidopsis*, affects the expression of mitochondrial *NAD1*, *RPL2*, *NAD9*, and *MATR* genes. *Arabidopsis* PGN mutant lines are susceptible to ABA and salt stress, and to necrotrophic fungal pathogen infections (Laluk et al., 2011).

Although the functional role of these organellar RBPs are still not fully understood, most of the currently reported stress-responsive organellar RBPs are involved in intron splicing of key genes or rRNA processing during organellar biogenesis under normal or stress conditions. Further research is required to identify other novel organellar RBPs and their target RNAs, and to uncover the mechanisms underlying their RNA chaperone function. Such knowledge will greatly enrich our understanding of how post-transcriptional gene regulation within organelles interfaces with normal plant growth and development and during stress.

## RNA-Binding Proteins Meet Stress Granules

A consequence of translational suppression under adverse environment is the sequestration of mRNA transcripts into aggregates of cytoplasmic RNA-protein complexes as stress granules (SGs) (Kedersha et al., 1999; Weber et al., 2008; Buchan and Parker, 2009). SGs are one type of cytoplasmic membrane-free structures mainly composed of polyadenylated mRNA transcripts together with translation initiation factors, the 40S ribosomal subunit, and RBPs (Chantarachot and Bailey-Serres, 2017). The formation and assembly of SGs are reversible, allowing the temporary storage of mRNAs in SGs under adverse conditions. Once released from SGs, mRNAs can be selectively sorted to the degradation pathway or re-enter the translational cycle (Lee, 2012). Thus, SGs functionally connect with two other cytoplasmic mRNP complex structures, *i.e.*, polysomes for active translation and processing bodies (PBs) for potential decay. Collectively, they form a triangular control hub of dynamic mRNA balance (Kedersha and Anderson, 2002; Brengues et al., 2005; Chantarachot and Bailey-Serres, 2017).

The evolutionary conserved SGs are highly dynamic organelles in eukaryotes. Although they were discovered more than 100 years ago (Miller, 1900), the nature of the aggregates, the mechanism of formation, and the dynamics of their compositions still remain elusive. Due to technical limitations, our understanding of plant SGs are derived mainly from yeast and mammalian studies. Maruri-López et al. (2019) provided a comprehensive review about the formation, assembly, disassembly and components of plant SGs with extended information from yeast and mammalian system. In plants, while a variety of stress conditions such as heat, salt, hypoxia and darkness, inhibition of oxidative phosphorylation, and hormone treatments can trigger the formation of SGs (Weber et al., 2008; Pomeranz M.C. et al., 2010; Sorenson and Bailey-Serres, 2014; Yan et al., 2014; Gutierrez-Beltran et al., 2016; Jang et al., 2020), the function and formation of plant SGs are best understood under heat and hypoxia stress.

Current knowledge suggests that SGs are formed *via* liquid-liquid phase separation (LLPS) of mRNP complexes and grow through a nucleation process with a core formation by the co-assembly of essential proteins. While the protein composition in SGs is heterogeneous and the protein components vary greatly according to the different stresses (Buchan et al., 2013; Mahboubi and Stochaj, 2017), emerging evidences have suggested the crucial role of RBPs to drive LLPS induced SG formation (Han et al., 2012; Kato et al., 2012; Molliex et al., 2015; Wheeler et al., 2016; Maruri-López et al., 2019). The LLPS process is considered to be highly dependent on the polymerization of low-complexity domain (LCD)-containing proteins (Han et al., 2012; Kato et al., 2012) that tends to be intrinsically disordered proteins (IDPs). While low-complexity domains are often observed in RNA and DNA binding proteins (Hennig et al., 2015; Chakrabortee et al., 2016; Ntountoumi et al., 2019), several mammalian RBPs such as fused in sarcoma (FUS) (Bosco et al., 2010), heterogeneous nuclear ribonucleoprotein A1 (hnRNPA1) (Molliex et al., 2015) and T-cell restricted intracellular antigen-1 (TIA-1) (Ding et al., 2021), polymerize *via* their low complexity domains and drive the transition of LLPS into SGs (Lin et al., 2015; Molliex et al., 2015; Boeynaems et al., 2018; Luo et al., 2018). In plants, a recent study reveals that two *Arabidopsis* glycine-rich RNA-binding proteins RBGD 2 and 4 undergo LLPS *in vitro* and accumulate into heat-induced SGs (Zhu et al., 2022). This process is driven by low complexity domains located in their C-termini where tyrosine residues are required to mediate RBGD 2/4 LLPS both *in vitro* and *in vivo* (Zhu et al., 2022). LLPS status is a reversible phenomenon with an equilibrium between polymerization and depolymerization (Kato et al., 2012; Li et al., 2012), which may directly contribute to the dynamic control of SG assembly and disassembly. Thus, RBPs are essential modulators of SG formation during stress response.

In addition to RBGD 2/4, several RBPs have been found to serve as core components and scaffolds to selectively sequester RNA transcripts and recruit other factors to mediate the formation, growth, assembly and stability of plant SGs (Niewidok et al., 2018; Duan et al., 2019). While their LLPS properties have not been extensively investigated, most of the RBPs specifically recognize and sequester target RNA transcripts to SGs. Here, we summarize the regulatory roles of specific RBPs in SG formation in plants.

Current evidence suggests that oligouridylate binding protein 1 (UBP1) and RNA-binding protein 45/47b (RBP45/47) family proteins are the core components of SGs. Both proteins contain three RRM domains and show high homology to TIA-1 (T-cell intracellular antigen 1) and TIAR (TIA-1 related protein), two proteins essential for human SG assembly (Kedersha et al., 1999; Gilks et al., 2005). They exhibit dynamic localization behavior shuttling between the cytoplasm and nucleus under normal conditions, but relocate to cytoplasmic SG foci under stress (Gilks et al., 2005). Thus, they are used as marker proteins to locate and visualize plant SGs (Sorenson and Bailey-Serres, 2014). In *Arabidopsis*, the UBP1 family contains 3 members, AtUBP1a, AtUBP1b and AtUBP1c. All are found to reversely form SGs upon heat stress (Sorenson and Bailey-Serres, 2014; Chau et al., 2016). Among them, overexpression of *UBP1b* induces the expression of 117 genes and enhances heat tolerance (Chau et al., 2016). A hypothesis derived from RNA decay analysis suggests that UBP1b SGs protect stress-related mRNAs from degradation during heat stress (Chau et al., 2016). *UBP1a* and *UBP1c* are also reported to respond low-oxygen stress (Sorenson and Bailey-Serres, 2014). UBP1c normally interacts with U-rich 3′UTR under non-stress conditions. During hypoxia, however, UBP1c prefers to bind non U-rich mRNAs and sequesters them into SGs (Sorenson and Bailey-Serres, 2014). When subjected to re-oxygenation, UBP1c SGs rapidly disassemble and release the stabilized mRNA to form polysome complexes (Sorenson and Bailey-Serres, 2014). Hence, UBP1 may function as molecular switch that selectively associates with target mRNAs and dynamically regulates SG assembly.

RNA-binding protein 45 and RBP47 family proteins usually associate with poly(A) + RNA (need to check format) as they participate in pre-mRNA maturation in the nucleus (Lorković et al., 2000). These RBPs relocate to SG foci in the cytoplasm when exposed to heat, salt and hypoxia (Weber et al., 2008; Yan et al., 2014; Gutierrez-Beltran et al., 2016). RBP47 was found to co-localize and behave identically with UBP1, suggesting they may play a similar role during stress response (Weber et al., 2008). RBP47b was reported to interact with other polyadenylate-binding proteins (PABPs), such as PABP2, PABP4, PABP5, and PABP8 (Kosmacz et al., 2019). PABP2 is also required for SG aggregation and used as a marker protein to visualize SGs. In addition, RBP47b was found to interact with the small molecule 2′, 3′-cAMP during SG formation under heat stress (Kosmacz et al., 2018), and recruit angustifolia protein (AN) to assemble SGs under high temperature, salt, osmotic and hypoxia stress conditions (Hemal and Martin, 2017). These observations suggest that RBP47 has a specific function, yet to be identified, in SG formation.

Tudor-SN (tudor staphylococcal nuclease) is a common SG protein found in mammals, yeast and plants (Sorenson and Bailey-Serres, 2014). Tudor-SN is an evolutionarily conserved RBP characterized by four complete staphylococcal nuclease (SN) domains at the N-terminal end, and a Tudor domain followed by a partial SN domain at the C terminus (Gutierrez-Beltran et al., 2016). Tudor-SNs was initially discovered as a transcriptional co-activator (Yang et al., 2006), but participates in a wide variety of activities in the nucleus, *e.g., in vitro* spliceosome assembly in *Drosophila* (Pham et al., 2004), and in the cytoplasm, *e.g.*, serving as a cytoskeleton-associated RNA-binding activity and component of RNA transport in rice (Wang et al., 2008; Chou et al., 2017). Under salt and heat stress, *Arabidopsis* lines harboring mutations in the Tudor-SN genes, *tsn1* and *tsn 2*, exhibit severe defects in seed germination, seedling growth, survival, and adaptability (Dit Frey et al., 2010; Yan et al., 2014; Gutierrez-Beltran et al., 2016). Further transcriptome and mRNA decay analyses of the mutants indicate the instability of its target transcripts and induce the assembly of translationally inactive ribonucleoparticles in the cytoplasm (Dit Frey et al., 2010). Tudor-SN are localized in heat-stressed induced SGs (Yan et al., 2014; Gutierrez-Beltran et al., 2016), together with other SG relevant proteins such as PAB4, HSP70, and RBP47b (Gutierrez-Beltran et al., 2021; Maruri-López et al., 2021). Moreover, the presence of Tudor-SN and SG formation are both required for activation of heat-induced SNF1-related protein kinase 1 (SnRK1) (Gutierrez-Beltran et al., 2021), an ortholog of the mammalian AMP-activated protein kinase (AMPK) and key regulator of TOR (target of rapamycin) (Shaw, 2009; Leene et al., 2019). Given the essential roles of SnRK1 and TOR proteins as integrators of transcriptional networks in stress and energy signaling (Baena-González et al., 2007; Belda-Palazón et al., 2020), TSN may engage with SG formation to activate stress-induced AMPK/SNF1/SnRK1 signaling. A recent study from *Arabidopsis* reveals that Tudor-SN itself is a highly disordered protein, and can act as a IDP to serve as a scaffold to recruit approximately 30% of its interacting proteins, forming a large IDP pool, to *de novo* induce stress granules upon stress perception (Gutierrez-Beltran et al., 2021). Taken together, in addition to its participation in regulating specific mRNAs and stress signaling, TSN may act as a docking platform to promote SG formation under stress condition.

Ras GTP SH3 domain binding proteins (G3PBs) are also associated with stress response and SG formation. G3BPs are usually characterized by the presence of a nuclear transport factor 2 (NTF2) like domain at the N-terminus, an RRM domain, and an arginine-glycine rich (RGG) region at the C-terminus with acid-rich and proline-rich (PXXP) regions in the center (Tourriere et al., 2001; Abulfaraj et al., 2021). G3BP members from *Arabidopsis* respond to high light, heat, salt and oxidative stress, although, unlike the other stress conditions, their expression is suppressed under oxidative stress (Abulfaraj et al., 2021). A recent study showed that all eight AtG3BPs are located in stress granule-like structures after heat treatment (Abulfaraj et al., 2021; Reuper et al., 2021). In human cells, the binding of G3BPs to 40S ribosomes *via* their RGG domain is required for stress granule condensation (Kedersha et al., 2016). This process is controlled by Caprin1 and USP10, where Caprin1 binding to G3BP promotes SG formation, whereas USP10 binding inhibits SG formation. Thus, G3BP may act as a switch to regulate the formation of SGs *via* its interaction with Caprin1 or USP10. In *Arabidopsis*, AtG3BPs is found to interact with AtUBP-24, a homolog of the human USP10, suggesting that plant G3BPs may play a similar role in SG formation (Reuper et al., 2021).

Both SGs and PBs are membrane-less cytoplasmic foci to sequester repressed mRNA. While PBs are distinct from SGs in possessing RNA-decapping and -degradation machineries, PBs and SGs are compositionally linked in sharing common components. This view is supported by the dual localization of RBPs in SGs and PBs, which also suggest the involvement of RBPs in the selective sorting of transcripts for degradation or storage. One common RBP activity found in SGs and PBs are the TZF proteins. They typically contain two zinc-binding CCCH motifs arranged in tandem and an Arg-rich motif upstream of the TZF motifs (Bogamuwa and Jang, 2014). *Arabidopsis* TZFs play diverse roles in plant growth and development, and respond to salt, drought, cold and oxidative stress (Bogamuwa and Jang, 2014; Han et al., 2021). AtTZF1 was found to shuttle between the nucleus and cytoplasmic PBs under normal condition, but predominantly target to SG-like foci during heat stress (Pomeranz M. et al., 2010). The other three TZFs, AtTZF4, AtTZF5, and AtTZF6, were also found to physically interact with both SGs and PBs, along with MEDIATOR OF ABA-REGULATED DORMANCY1 and RESPONSIVE TO DEHYDRATION21A, during seed germination (Bogamuwa and Jang, 2014). In rice, OsTZF1, which is induced by drought, salt, abscisic acid, methyl jasmonate, and salicylic acid, localizes in cytoplasmic foci and its co-localization with SG and PB markers is enhanced under stress conditions (Jan et al., 2013). This is consistent with the human TZF family protein tristetraprolin (TTP), which shuttles between the nucleus and cytoplasm but is concentrated in SGs and PBs under stress conditions (Phillips et al., 2002). A more recent study reported that *Arabidopsis* DHH1/DDX6-like RNA helicases, RH6, RH8, and RH12, physically associate with both PBs and SGs and co-localize with their marker proteins DCP2 and UBP1C, respectively (Chantarachot et al., 2020). Although SGs and PBs share common RBPs, the specific roles of these RBPs in these membrane-less organelles remain unclear. The discovery of supramolecular complexes of SGs and PBs in tobacco mesophyll protoplasts (Weber et al., 2008), which may serve as sorting hub for PBs and SGs, adds another layer of mystery to the regulatory mechanism underlying the close relationship between SGs and PBs. Whether these common RBPs are the main determinant factors in determining mRNA fate and regulating the kinetic formation of SGs and PBs deserve further investigation in future.

## RNA-Binding Proteins Interplay With Abscisic Acid

Abscisic acid (ABA) has been called the stress hormone as it triggers plant stress responses and regulates complex communication among different stress signals (Mehrotra et al., 2014). When adverse environmental conditions appear, especially under osmotic stress induced by drought or salinity, ABA biosynthesis is significantly enhanced. In turn, the elevated ABA levels initiate signal transduction by binding to its receptor, which leads to a variety of plant responses including stomatal closure, changes in gene expression, and adaptive physiological responses (Ng et al., 2014; Sah et al., 2016). ABA also plays essential roles in many other cellular processes, such as seed production and germination, vegetative growth, and modulation of root architecture (Harris, 2015; Benderradji et al., 2021).

Along with the discovery of RBPs in stress response, considerable effort also reveals a close connection between RBPs and ABA. One important example of a RBP closely related to ABA is the ABA-activated protein kinase (AAPK)-interacting protein 1 (AKIP1), a heterogeneous nuclear ribonucleoprotein (hnRNP) initially identified in *Vicia faba* (Li et al., 2000, 2002). ABA induces the phosphorylation of AKIP1, which activates its interaction with mRNAs to form subnuclear foci reminiscent of nuclear speckles under ABA treatment (Li et al., 2002). A close homolog of AKIP1 in *Arabidopsis* is the poly(U)-Binding Associated protein (UBA2a), which also showed similar behavior of relocation to nuclear speckles in response to exogenous ABA and drought stress (Riera et al., 2006; Bove et al., 2008). The UBA family proteins, including UBA1 and UBA2 families, are also called UBP1-associated proteins due to their direct interaction with UBP1. UBP1, UBA1a, and UBA2a are nuclear proteins and may act as a complex to recognize U-rich region in 3′-UTRs enabling mRNA maturation and stability in the nucleus (Lambermon et al., 2002; Riera et al., 2006; Wachter et al., 2012) during ABA-dependent stress response.

As shown in Table 1, the majority of the stress associated RBPs respond to both ABA and stress treatment, suggesting these RBPs function in an ABA-dependent pathway during stress. ABA reduces the expression of *AtRZ-1a* (Kim et al., 2005), DEAD box RNA helicase genes such as the *LOS4* (low expression of osmotically responsive genes 4), and *STRS1* and *STRS2* (STRESS RESPONSE SUPPRESSOR1 and 2) (Gong et al., 2005; Kant et al., 2007). While exogenous ABA inhibits seed germination of the *AtRZ-1a* overexpression line, it promotes the germination of mutant seeds under salt or drought stress conditions (Kim Y.O. et al., 2007). Likewise, a mutation in the DEAD box RNA helicase genes confers an ABA hypersensitive phenotype and improves tolerance to multiple abiotic stresses including cold, salt, osmotic, and heat (Gong et al., 2005; Kant et al., 2007). These results indicate that these genes negatively regulate ABA-dependent plant stress response. Additionally, the mRNA cap-binding protein *ABH1* (abscisic acid hypersensitive 1), the Sm-like small nuclear ribonucleoprotein SAD1 (supersensitive to ABA and drought 1), and the double-stranded RNA-binding protein HYL1 (hyponastic leaves 1) have also been identified as negative regulators of ABA-dependent seed germination and drought tolerance (Lu and Fedoroff, 2000; Xiong et al., 2001; Hugouvieux et al., 2002; Kuhn, 2003; Hg et al., 2005).

The *Arabidopsis* SR45 protein may also function as a negative regulator of ABA as well as glucose signaling during seedling development (Carvalho et al., 2016). Palusa et al. (2007) performed a comprehensive analysis of alternative splicing pattern of SR proteins in *Arabidopsis* under hormone and stress treatments. They found that most of the SR genes underwent differential alternative splicing patterns under ABA treatment or salt stress (Palusa et al., 2007). Although their function as a negative regulator in ABA and stress responses is largely unknown, SR proteins are thought to play crucial roles in multiple steps of nuclear RNA processing and mRNA export and thus affect the expression of known stress-responsive genes and ABA relevant signal molecules to increase plant sensitivity to ABA and stress (Gong et al., 2005). For example, the *ABH1* defective mutant showed mis-expression of the crucial ABA signaling molecule AtPP2C (Hugouvieux et al., 2001), a known negative regulator in ABA signaling, which may contribute to the ABA hypersensitive phenotype in the mutant.

On the other hand, the expression of some RBPs are positively associated with ABA treatment. For example, BrRZ1, 2 and 3 (Park et al., 2017), BrCSDP3 (Choi et al., 2015), and BrRH22 (Nawaz et al., 2018) from *Brassica napus* positively respond to ABA induction. Likewise, the expression of several GRPs, *OsGRP3* (Shim et al., 2021), *NtGRP1* (Lee et al., 2009; Khan et al., 2013), *MsGRP* (Long et al., 2013), *LpGRP1* (Shinozuka et al., 2006), and *NgRBP* (Huang et al., 2019), increase under treatment of ABA (Table 1). The *Arabidopsis* nucleocytoplasmic AtTZF1 acts as a positive regulator of ABA and sugar responses and its overexpression enhances plant tolerance to cold and drought stresses (Lin et al., 2011). Analysis from microarray indicate that over-expression of AtTZF1 down-regulate the expression of GA-Stimulated *Arabidopsi*s 6 (GASA6), a GA-inducible and ABA-repressible peptide hormone, thus functioning as an upstream regulator to modulate ABA signaling (Lin et al., 2011).

Organellar-localized proteins play distinct roles in the plant’s response to ABA. A recent study (Kwanuk et al., 2019) found that the mitochondria-localized *Arabidopsis* CFM9, a CRM domain-containing protein, positively regulates *Arabidopsis* seed germination and seedling growth in the presence of ABA and stress. The loss-of-function mutant of the chloroplast-localized RH3, which is involved in the splicing of *ndhA* and *ndhB* introns, is hypersensitive to ABA (Gu et al., 2014). Mutation of the chloroplast-localized PPR protein GENOMES UNCOUPLED1 (GUN1) confers slow-growth phenotype under ABA treatment in *Arabidopsis* (Cottage et al., 2010). While mediating a plastid to nucleus retrograde signaling pathway during chloroplast biogenesis, GUN1 is reported to regulate the expression of *LHCB1* (Cottage et al., 2010) and the functionally related cold and ABA responsive AtRH50 that is require for the maturation of *23S* and *4.5S rRNAs* (Paieri et al., 2018). The rice WSL, which is involved in the splicing of chloroplast *rpl2* introns, shows enhanced seed germination and seedling growth in response to ABA, owing to its reduced translation efficiency (Tan et al., 2014). *Arabidopsis* ABO5 and ABO8, which are involved in the splicing of mitochondrial *nad2* intron3 and *nad4* intron3, have been shown to have increased sensitivity to ABA under post-germination and root growth phase by accumulating reactive oxygen species (ROS) in the mitochondria (Liu et al., 2010; Yang et al., 2014). Chloroplast-targeted SRRP1, which has two S1 domains, is involved in intron splicing of chloroplast tRNAs. Loss of gene function decreases plant sensitivity to ABA and impairs the splicing of the chloroplast *trnL* intron and processing of *5S rRNA* in the presence of ABA (Gu et al., 2015).

Irrespective of whether they are negative or positive regulators in ABA signaling, the current studies reveal a dual relationship between RBPs and the ABA signaling pathway. That is, ABA can significantly affect the expression of RBPs and, in turn, post-transcriptional control of gene expression. Hence, RBPs are critical components for ABA signaling. Further identification and characterization of the direct targets of these RBPs will be helpful to elucidate the molecular mechanisms underlying ABA signaling and stress response.

Although there is no evidence that stress-induced ABA signaling pathway has a direct relationship with stress granules, the association of RBPs common to both ABA signaling pathway and stress granule formation infers a connection. It was reported that elevated cytoplasmic concentrations of hnRNPA1, hnRNPA2 and FUS, RNA-binding proteins that contain low complexity domains, resulted in an increased assembly of stress granules in human HeLa cells (Molliex et al., 2015). *In vitro* cell free study of RNA granule formation suggest that high concentrations of low complexity domain-containing proteins promote LLPS process required for SG formation (Han et al., 2012; Kato et al., 2012; Zhu et al., 2022). Thus, the concentration of cytoplasmic RBPs may have direct effect to trigger LLPS of RBPs and in turn, SG nucleation within the cell. Similar situation may occur in plant cells. Indeed, in the case study of OsTZF1, ABA treatment enhanced the formation of OsTZF1 associated stress granule-like foci in rice root cells (Jan et al., 2013). Given that ABA treatment promotes the expression of OsTZF1, the enhanced appearance of SG-like foci may be due to the triggering of LLPS formation mediated by high concentrations of OsTZF1. Although further study is required, we hypothesize that ABA treatment may trigger the formation of SGs through increasing the concentration of ABA-responsive RBPs.

It is likely that not all of the RBPs involved in stress responses interplay with ABA (Table 1) *i.e*., the regulatory role of some RBPs can be ABA-independent. For example, overexpression of AtGRP2 does not accelerate *Arabidopsis* seed germination and seedling growth following addition of abscisic acid (ABA) when compared to wild-type plants (Kim et al., 2010c), implying that AtGRP2 affects seed germination *via* an ABA-independent pathway. Another example is the nuclear DEAD-box RH protein AtRH17. When overexpressed in *Arabidopsis*, the transgenic lines display tolerance to salt stress (Nguyen et al., 2018). Based on transcriptome analysis, however, no changes are observed between ABA-dependent and ABA-independent pathways in the transgenic lines (Nguyen et al., 2018), implying the possible existence of an unidentified stress-responsive pathway.

## Future Direction

Along with the improvement of high-throughput -omics techniques combined with protein-RNA interaction technology, we are now beginning to understand the diverse biological roles of RBPs in plant growth and development, and during plant stress. Due to their modular structures, RBPs are multifaceted in mediating the fate of RNA through post-transcriptional gene regulation. Although a growing body of evidence shows a close association of RBPs during plant stress tolerance, SGs formation, and ABA signaling, our understanding of RBPs in these processes remain extremely limited and many knowledge gaps remain to be resolved. These include the specific RNAs targeted by these RBPs and their interacting protein partners during normal plant growth and development as well as under stress, the functional roles of RBPs and their interacting protein partners during the dynamic interchange of SGs with PBs and active polysomes, and the underlying mechanism of RBPs with the ABA transduction signaling pathway. Applications using RNA immunoprecipitation (RIP) coupled with high-throughput sequencing (RIP-seq) in combination with crosslinking (CLIP-seq) may help to elucidate a more detailed landscape of RBPs and their specific target RNAs. The newly developed technologies in mammals, such as targets of RNA-binding protein identified by editing (TRIBE) and RNA tagging (Aoife et al., 2016), may also be used as alternative approaches to identify the genome-wide RBP targets. The employment of high-resolution microscopy techniques assisted with cell type-specific isolation and subcellular fractionation can provide unprecedented information to determine the precise functions of RBPs in the nucleus, cytosol, and other organelles and reveal their possible function in SG formation. The functional characterization of individual RBP will also be extremely important to enrich our understanding about RBPs in stress response. Precise gene editing and knockout tools such CRISPR/Cas9 will provide a promising approach to characterize the functions of individual RBPs under abiotic stress conditions.

## Author Contributions

YY, TO, and LT designed and wrote the manuscript. YY, JG, and YT collected data and prepared Table 1. All authors have read and proved the final version of the manuscript.

## Conflict of Interest

The authors declare that the research was conducted in the absence of any commercial or financial relationships that could be construed as a potential conflict of interest.

## Publisher’s Note

All claims expressed in this article are solely those of the authors and do not necessarily represent those of their affiliated organizations, or those of the publisher, the editors and the reviewers. Any product that may be evaluated in this article, or claim that may be made by its manufacturer, is not guaranteed or endorsed by the publisher.

## References

[B1] Abulfaraj A. A. Hirt H. Rayapuram N. (2021). G3BPs in plant stress. *Front. Plant Sci.* 12:680710. 10.3389/fpls.2021.680710 34177995 PMC8222905

[B2] Abulfaraj A. A. Mariappan K. Bigeard J. Manickam P. Blilou I. Guo X. (2018). The *Arabidopsis* homolog of human G3BP1 is a key regulator of stomatal and apoplastic immunity. *Life Sci.* 1:e201800046. 10.26508/lsa.201800046 30456348 PMC6238584

[B3] Albà M. M. Pagès M. (1998). Plant proteins containing the RNA-recognition motif. *Trends Plant Sci.* 3 15–21. 10.1016/S1360-1385(97)01151-5

[B4] Ambrosone A. Costa A. Martinelli R. Massarelli I. De Simone V. Grillo S. (2010). Differential gene regulation in potato cells and plants upon abrupt or gradual exposure to water stress. *Acta Physiol. Plant* 33 1157–1171. 10.1007/s11738-010-0644-1

[B5] Aoife C. M. Rahman R. Jin H. Shen J. L. Fieldsend A. Luo W. F. (2016). TRIBE: hijacking an RNA-editing enzyme to identify cell-specific targets of RNA-binding proteins. *Cell* 165 742–753. 10.1016/j.cell.2016.03.007 27040499 PMC5027142

[B6] Asakura Y. Barkan A. (2007). A CRM domain protein functions dually in group I and group II intron splicing in land plant chloroplasts. *Plant Cell* 19 3864–3875. 10.1105/tpc.107.055160 18065687 PMC2217638

[B7] Bach-Pages M. Homma F. Kourelis J. Kaschani F. Mohammed S. Kaiser M. (2020). Discovering the RNA-binding proteome of plant leaves with an improved RNA interactome capture method. *Biomolecules* 10:661. 10.3390/biom10040661 32344669 PMC7226388

[B8] Baena-González E. Rolland F. Thevelein J. M. Sheen J. (2007). A central integrator of transcription networks in plant stress and energy signalling. *Nature* 448 938–942. 10.1038/nature06069 17671505

[B9] Belda-Palazón B. Adamo M. Valerio C. Ferreira L. J. Confraria A. Reis-Barata D. (2020). A dual function of SnRK2 kinases in the regulation of SnRK1 and plant growth. *Nat. Plants* 6 1345–1353. 10.1038/s41477-020-00778-w 33077877

[B10] Benderradji L. Saibi W. Brini F. (2021). Role of ABA in overcoming environmental stress: sensing, signaling and crosstalk. *Ann. Agric. Crop Sci.* 6:1070.

[B11] Boeynaems S. Alberti S. Fawzi N. L. Mittag T. Polymenidou M. Rousseau F. (2018). Protein phase separation: a new phase in cell biology. *Trends Cell Biol.* 28 420–435. 10.1016/j.tcb.2018.02.004 29602697 PMC6034118

[B12] Bogamuwa S. Jang J. C. (2014). Tandem CCCH zinc finger proteins in plant growth, development and stress response. *Plant Cell Physiol.* 55:1367. 10.1093/pcp/pcu074 24850834

[B13] Bogamuwa S. Jang J. C. (2016). Plant tandem CCCH zinc finger proteins interact with ABA, drought, and stress response regulators in processing-bodies and stress granules. *PLoS One* 11:e0151574. 10.1371/journal.pone.0151574 26978070 PMC4792416

[B14] Bosco D. A. Lemay N. Ko H. K. Zhou H. Burke C. Kwiatkowski T. J. J. (2010). Mutant FUS proteins that cause amyotrophic lateral sclerosis incorporate into stress granules. *Hum. Mol. Genet.* 19 4160–4175. 10.1093/hmg/ddq335 20699327 PMC2981014

[B15] Bove J. Kim C. Y. Gibson C. A. Assmann S. M. (2008). Characterization of wound-responsive RNA-binding proteins and their splice variants in *Arabidopsis*. *Plant Mol. Biol.* 67 71–88. 10.1007/s11103-008-9302-z 18278441

[B16] Brengues M. Teixeira D. Parker R. (2005). Movement of eukaryotic mRNAs between polysomes and cytoplasmic processing bodies. *Science* 310 486–489. 10.1126/science.1115791 16141371 PMC1863069

[B17] Buchan J. Kolaitis R. M. Taylor J. Parker R. (2013). Eukaryotic stress granules are cleared by autophagy and Cdc48/VCP function. *Cell* 153 1461–1474. 10.1016/j.cell.2013.05.037 23791177 PMC3760148

[B18] Buchan J. R. Parker R. (2009). Eukaryotic stress granules: the ins and outs of translation. *Mol. Cell* 36 932–941. 10.1016/j.molcel.2009.11.020 20064460 PMC2813218

[B19] Cao S. Q. Jiang L. Song S. Y. Jing R. Xu G. S. (2006). AtGRP7 is involved in the regulation of abscisic acid and stress responses in *Arabidopsis*. *Cell. Mol. Biol. Lett.* 11 526–535. 10.2478/s11658-006-0042-2 17001447 PMC6275784

[B20] Carvalho R. F. Szakonyi D. Simpson C. G. Barbosa I. C. R. Brown J. W. S. Baena-González E. (2016). The *Arabidopsis* SR45 splicing factor, a negative regulator of sugar signaling, modulates SNF1-related protein kinase 1 stability. *Plant Cell* 28 1910–1925. 10.1105/tpc.16.00301 27436712 PMC5006706

[B21] Chaikam V. Karlson D. (2008). Functional characterization of two cold shock domain proteins from Oryza sativa. *Plant Cell Environ.* 31 995–1006. 10.1111/j.1365-3040.2008.01811.x 18397370

[B22] Chakrabortee S. Kayatekin C. Newby G. A. Mendillo M. L. Lancaster A. Lindquist S. (2016). Luminidependens (LD) is an *Arabidopsis* protein with prion behavior. *Proc. Natl. Acad. Sci. U.S.A.* 113 6065–6070. 10.1073/pnas.1604478113 27114519 PMC4889399

[B23] Chantarachot T. Bailey-Serres J. (2017). Polysomes, stress granules and processing bodies: a dynamic triumvirate controlling cytoplasmic mRNA fate and function. *Plant Physiol.* 176 254–269. 10.1104/pp.17.01468 29158329 PMC5761823

[B24] Chantarachot T. Sorenson R. S. Hummel M. Ke H. Y. Kettenburg A. T. Chen D. (2020). DHH1/DDX6-like RNA helicases maintain ephemeral half-lives of stress-response mRNAs. *Nat. Plants* 6 675–685. 10.1038/s41477-020-0681-8 32483330

[B25] Chau N. C. Kentaro N. Akihiro M. Shuhei K. Yukio K. Kiminori T. (2016). Oligouridylate binding protein 1b plays an integral role in plant heat stress tolerance. *Front. Plant Sci.* 7:853. 10.3389/fpls.2016.00853 27379136 PMC4911357

[B26] Choi M. J. Park Y. R. Park S. J. Kang H. S. (2015). Stress-responsive expression patterns and functional characterization of cold shock domain proteins in cabbage (*Brassica rapa*) under abiotic stress conditions. *Plant Physiol. Biochem.* 96 132–140. 10.1016/j.plaphy.2015.07.027 26263516

[B27] Chou H. L. Tian L. Kumamaru T. Okita T. W. (2017). Multifunctional RNA binding protein OsTudor-SN in storage protein mRNA transport and localization. *Plant Physiol.* 175 1608–1623. 10.1104/pp.17.01388 29084903 PMC5717745

[B28] Ciuzan O. Lazar S. L. Lung M. L. Pop O. L. Pamfil D. (2015). Involvement of the glycine-rich RNA-binding proteins (GRP) in plant abiotic stress response: a comparison between GRP 2 and GRP 7. *Bull. Univ. Agric. Sci. Vet. Med. Cluj Napoca Hort.* 72 61–67. 10.15835/buasvmcn-hort:10912

[B29] Cottage A. Mott E. K. Kempster J. A. Gray J. C. (2010). The *Arabidopsis* plastid-signalling mutant gun1 (genomes uncoupled1) shows altered sensitivity to sucrose and abscisic acid and alterations in early seedling development. *J. Exp. Bot.* 61 3773–3786. 10.1093/jxb/erq186 20605896 PMC2921207

[B30] Cruz J. Kressler D. Linder P. (1999). Unwinding RNA in *Saccharomyces cerevisiae*: DEAD-box proteins and related families. *Trends Biochem. Sci.* 24 192–198. 10.1016/S0968-0004(99)01376-6 10322435

[B31] Ding X. F. Gu S. Y. Xue S. Luo S. Z. (2021). Disease-associated mutations affect TIA1 phase separation and aggregation in a proline-dependent manner. *Brain Res.* 1768:147589. 10.1016/j.brainres.2021.147589 34310938

[B32] Dinh S. N. Park S. J. Han J. H. Kang H. (2019). A chloroplast-targeted S1 RNA-binding domain protein plays a role in *Arabidopsis* response to diverse abiotic stresses. *J. Plant Biol.* 62 74–81. 10.1007/s12374-018-0325-y

[B33] Dit Frey N. F. Muller P. Jammes F. Kizis D. Leung J. Perrot-Rechenmann C. (2010). The RNA binding protein Tudor-SN is essential for stress tolerance and stabilizes levels of stress-responsive mRNAs encoding secreted proteins in *Arabidopsis*. *Plant Cell* 22 1575–1591. 10.1105/tpc.109.070680 20484005 PMC2899877

[B34] Duan Y. Du A. Gu J. Duan G. Wang C. Gui X. (2019). PARylation regulates stress granule dynamics, phase separation, and neurotoxicity of disease-related RNA-binding proteins. *Cell Res.* 29 233–247. 10.1038/s41422-019-0141-z 30728452 PMC6460439

[B35] Flores A. F. Sachetto-Martins G. (2007). Blooming time for plant glycine-rich proteins. *Plant Signal. Behav.* 2 386–387. 10.4161/psb.2.5.4262 19704608 PMC2634221

[B36] Francesco D. Giulia P. Gianni D. Federica B. (2011). S1 ribosomal protein and the interplay between translation and mRNA decay. *Nucleic Acids Res.* 39 7702–7715. 10.1093/nar/gkr417 21685451 PMC3177188

[B37] Gilks N. Kedersha N. Ayodele M. Shen L. Stoecklin G. Dember L. M. (2005). Stress granule assembly is mediated by prion-like aggregation of TIA-1. *Mol. Biol. Cell* 15 5383–5398. 10.1091/mbc.E04-08-0715 15371533 PMC532018

[B38] Glisovic T. Bachorik J. L. Yong J. Dreyfuss G. (2008). RNA-binding proteins and post-transcriptional gene regulation. *FEBS Lett.* 582 1977–1986. 10.1016/j.febslet.2008.03.004 18342629 PMC2858862

[B39] Gómez J. Sánchez-Martínez D. Stiefel V. Rigau J. Puigdomènech P. Pagès M. (1988). A gene induced by the plant hormone abscisic acid in response to water stress encodes a glycine-rich protein. *Nature* 334 262–264. 10.1038/334262a0 2969461

[B40] Gong X. D. Su Q. Q. Lin D. Z. Jiang Q. Xu J. L. Zhang J. H. (2014). The rice OsV4 encoding a novel pentatricopeptide repeat protein is required for chloroplast development during the early leaf stage under cold stress. *J. Integr. Plant Biol.* 56 400–410. 10.1111/jipb.12138 24289830

[B41] Gong Z. H. Dong C. H. Lee H. J. Zhu J. H. Xiong L. M. Gong D. M. (2005). A DEAD box RNA helicase is essential for mRNA export and important for development and stress responses in *Arabidopsis*. *Plant Cell* 17 256–267. 10.1105/tpc.104.027557 15598798 PMC544503

[B42] Gu L. Jung H. J. Kim B. M. Xu T. Lee K. Kim Y. O. (2015). A chloroplast-localized S1 domain-containing protein SRRP1 plays a role in *Arabidopsis* seedling growth in the presence of ABA. *J. Plant Physiol.* 189 34–41. 10.1016/j.jplph.2015.10.003 26513458

[B43] Gu L. Tao X. Lee K. Lee K. H. Kang H. (2014). A chloroplast-localized DEAD-box RNA helicaseAtRH3 is essential for intron splicing and plays an important role in the growth and stress response in *Arabidopsis thaliana*. *Plant Physiol. Biochem.* 82 309–318. 10.1016/j.plaphy.2014.07.006 25043599

[B44] Gutierrez-Beltran E. Denisenko T. V. Zhivotovsky B. Bozhkov P. V. (2016). Tudor staphylococcal nuclease: biochemistry and functions. *Cell Death Differ.* 23 1739–1748. 10.1038/cdd.2016.93 27612014 PMC5071578

[B45] Gutierrez-Beltran E. Elander P. H. Dalman K. Dayhoff G. W. II Moschou P. Uversky V. N. (2021). Tudor staphylococcal nuclease is a docking platform for stress granule components and is essential for SnRK1 activation in *Arabidopsis*. *EMBO J.* 40:e105043. 10.15252/embj.2020105043 34287990 PMC8447601

[B46] Gutierrez-Beltran E. Moschou P. N. Smertenko A. P. Bozhkov P. V. (2015). Tudor staphylococcal nuclease links formation of stress granules and processing bodies with mRNA catabolism in *Arabidopsis*. *Plant Cell* 27 926–943. 10.1105/tpc.114.134494 25736060 PMC4558657

[B47] Han G. Qiao Z. Li Y. Wang C. Wang B. (2021). The roles of CCCH zinc-finger proteins in plant abiotic stress tolerance. *Int. J. Mol. Sci.* 22:8327. 10.3390/ijms22158327 34361093 PMC8347928

[B48] Han J. H. Lee K. Lee K. H. Jung S. Jeon Y. Pai H. S. (2015). A nuclear-encoded chloroplast-targeted S1 RNA-binding domain protein affects chloroplast rRNA processing and is crucial for the normal growth of *Arabidopsis thaliana*. *Plant J.* 83 277–289. 10.1111/tpj.12889 26031782

[B49] Han T. W. Kato M. Xie S. Wu L. C. Mirzaei H. Pei J. (2012). Cell-free formation of RNA granules: bound RNAs identify features and components of cellular assemblies. *Cell* 149 768–779. 10.1016/j.cell.2012.04.016 22579282

[B50] Harris J. M. (2015). Abscisic acid: hidden architect of root system structure. *Plants* 4 548–572. 10.3390/plants4030548 27135341 PMC4844405

[B51] Hemal B. Martin H. (2017). ANGUSTIFOLIA, a plant homolog of CtBP/BARS localizes to stress granules and regulates their formation. *Front. Plant Sci.* 8:1004. 10.3389/fpls.2017.01004 28659951 PMC5469197

[B52] Hennig S. Kong G. Mannen T. Sadowska A. Kobelke S. Blythe A. (2015). Prion-like domains in RNA binding proteins are essential for building subnuclear paraspeckles. *J. Cell Biol.* 210 529–539. 10.1083/jcb.201504117 26283796 PMC4539981

[B53] Hg C. K. L. Toshinori K. Sona P. Ken-ichiro S. Assmann S. M. (2005). Abscisic acid induces rapid subnuclear reorganization in guard cells. *Plant Physiol.* 134 1327–1331. 10.1104/pp.103.034728 15084726 PMC419809

[B54] Horvath D. P. Olson P. A. (1998). Cloning and characterization of cold-regulated glycine-rich RNA-binding protein genes from leafy spurge (*Euphorbia esula* L.) and comparison to heterologous genomic clones. *Plant Mol. Biol.* 38 531–538. 10.1023/a:1006050208670 9747799

[B55] Huang C. K. Shen Y. L. Huang L. F. Wu S. J. Yeh C. H. Lu C. A. (2016). The DEAD-box RNA helicase AtRH7/PRH75 participates in pre-rRNA processing, plant development and cold tolerance in *Arabidopsis*. *Plant Cell Physiol.* 57 174–191. 10.1093/pcp/pcv188 26637537

[B56] Huang X. Yu R. Li W. J. Geng L. W. Jing X. L. Zhu C. X. (2019). Identification and characterisation of a glycine-rich RNA-binding protein as an endogenous suppressor of RNA silencing from *Nicotiana glutinosa*. *Planta* 249 1811–1822. 10.1007/s00425-019-03122-5 30840177

[B57] Hugouvieux V. Kwak J. M. Schroeder J. I. (2001). An mRNA cap binding protein, ABH1, modulates early abscisic acid signal transduction in *Arabidopsis*. *Cell* 106 477–487. 10.1016/S0092-8674(01)00460-3 11525733

[B58] Hugouvieux V. Murata Y. Joung J. J. Kwak J. M. Mackesy D. Z. Schroeder J. I. (2002). Localization, ion channel regulation, and genetic interactions during abscisic acid signaling of the nuclear mRNA cap-binding protein, ABH1. *Plant Physiol.* 130 1276–1287. 10.1104/pp.009480 12427994 PMC166648

[B59] Huh S. Paek K. H. (2014). APUM5, encoding a Pumilio RNA binding protein, negatively regulates abiotic stress responsive gene expression. *BMC Plant Biol.* 14:75. 10.1186/1471-2229-14-75 24666827 PMC3986970

[B60] Hunger K. Beckering C. L. Wiegeshoff F. Graumann P. L. Marahiel M. A. (2006). Cold-induced putative DEAD box RNA helicases CshA and CshB are essential for cold adaptation and interact with cold shock protein B in *Bacillus subtilis*. *J. Bacteriol.* 188 240–248. 10.1128/JB.188.1.240-248.2006 16352840 PMC1317592

[B61] Jacobs J. Kück U. (2011). Function of chloroplast RNA-binding proteins. *Cell. Mol. Life Sci.* 68 735–748. 10.1007/s00018-010-0523-3 20848156 PMC11115000

[B62] Jan A. Maruyama K. Todaka D. Kidokoro S. Abo M. Yoshimura E. (2013). OsTZF1, a CCCH-tandem zinc finger protein, confers delayed senescence and stress tolerance in rice by regulating stress-related genes. *Plant Physiol.* 161 1202–1216. 10.2307/41943540 23296688 PMC3585590

[B63] Jang G. J. Jang J. C. Wu S. H. (2020). Dynamics and functions of stress granules and processing bodies in plants. *Plants* 9:1122. 10.3390/plants9091122 32872650 PMC7570210

[B64] Jiang S. C. Mei C. Liang S. Yu Y. T. Lu K. Wu Z. (2015). Crucial roles of the pentatricopeptide repeat protein SOAR1 in *Arabidopsis* response to drought, salt and cold stresses. *Plant Mol. Biol.* 88 369–385. 10.1007/s11103-015-0327-9 26093896 PMC4486114

[B65] Kant P. Kant S. Gordon M. Shaked R. Barak S. (2007). Stress response suppressor1 and stress response suppressor2, two DEAD-Box RNA helicases that attenuate *Arabidopsis* responses to multiple abiotic stresses. *Plant Physiol.* 145 814–830. 10.1104/pp.107.099895 17556511 PMC2048787

[B66] Kato M. Han T. W. Xie S. Shi K. Du X. Wu L. C. (2012). Cell-free formation of RNA granules: low complexity sequence domains form dynamic fibers within hydrogels. *Cell* 149 753–767. 10.1016/j.cell.2012.04.017 22579281 PMC6347373

[B67] Kedersha N. Anderson P. (2002). Stress granules: sites of mRNA triage that regulate mRNA stability and translatability. *Biochem. Soc. Trans.* 30 963–969. 10.1042/BST0300963 12440955

[B68] Kedersha N. Panas M. Achorn C. A. Lyons S. Tisdale S. Hickman T. (2016). G3BP–Caprin1–USP10 complexes mediate stress granule condensation and associate with 40S subunits. *J. Cell Biol.* 212 845–860. 10.1083/jcb.201508028 27022092 PMC4810302

[B69] Kedersha N. L. Gupta M. Li W. Miller I. Anderson P. (1999). RNA-binding proteins TIA-1 and TIAR link the phosphorylation of eIF-2 alpha to the assembly of mammalian stress granules. *J. Cell Biol.* 147 1431–1442. 10.1083/jcb.147.7.1431 10613902 PMC2174242

[B70] Khan F. Sultana T. Deeba F. Naqvi S. M. (2013). Dynamics of mRNA of glycine-rich RNA-binding protein during wounding, cold and salt stresses in *Nicotiana tabacum*. *Pak. J. Bot.* 45 297–300. 10.1080/01904167.2012.759230

[B71] Kim J. S. Jung H. J. Lee H. J. Kim K. A. Goh C. H. Woo Y. M. (2008). Glycine-rich RNA-binding protein7 affects abiotic stress responses by regulating stomata opening and closing in *Arabidopsis thaliana*. *Plant J.* 55 455–466. 10.1111/j.1365-313X.2008.03518.x 18410480

[B72] Kim J. S. Park S. J. KwaK K. J. Kim Y. O. Kim J. Y. Song J. (2007). Cold shock domain proteins and glycine-rich RNA-binding proteins from *Arabidopsis thaliana* can promote the cold adaptation process in *Escherichia coli*. *Nucleic Acids Res.* 35 506–516. 10.1093/nar/gkl1076 17169986 PMC1802614

[B73] Kim J. Y. Kim W. Y. Kwak K. J. Oh S. H. Han Y. S. Kang H. (2010a). Glycine-rich RNA-binding proteins are functionally conserved in *Arabidopsis thaliana* and *Oryza sativa* during cold adaptation process. *J. Exp. Bot.* 61 2317–2325. 10.1093/jxb/erq058 20231330 PMC2877889

[B74] Kim J. Y. Kim W. Y. Kwak K. J. Oh S. H. Kang H. (2010b). Zinc finger-containing glycine-rich RNA-binding protein in *Oryza sativa* has an RNA chaperone activity under cold stress conditions. *Plant Cell Environ.* 33 759–768. 10.1111/j.1365-3040.2009.02101.x 20088860

[B75] Kim J. Y. Su J. P. Jang B. Jung C. H. Kang H. (2010c). Functional characterization of a glycine-rich RNA-binding protein 2 in *Arabidopsis thaliana* under abiotic stress conditions. *Plant J.* 50 439–451. 10.1111/j.1365-313X.2007.03057.x 17376161

[B76] Kim M. H. Sasaki K. Imai R. (2009). Cold shock domain protein 3 regulates freezing tolerance in *Arabidopsis thaliana*. *J. Biol. Chem.* 284 23454–23460. 10.1074/jbc.M109.025791 19556243 PMC2749119

[B77] Kim Y. O. Kim J. S. Kang H. (2005). Cold-inducible zinc finger-containing glycine-rich RNA-binding protein contributes to the enhancement of freezing tolerance in *Arabidopsis thaliana*. *Plant J.* 42 890–900. 10.1111/j.1365-313X.2005.02420.x 15941401

[B78] Kim Y. O. Pan S. Jung C.-H. Kang H. (2007). A zinc finger-containing glycine-rich RNA-binding protein, atRZ-1a, has negative impact on seed germination and seedling growth of *Arabidopsis thaliana* under salt or drought stress conditions. *Plant Cell Physiol.* 8 1170–1181. 10.1093/pcp/pcm087 17602187

[B79] Kosmacz M. Gorka M. Schmidt S. Luzarowski M. Moreno J. C. Szlachetko J. (2019). Protein and metabolite composition of *Arabidopsis* stress granules. *New Phytol.* 222 1420–1433. 10.1111/nph.15690 30664249

[B80] Kosmacz M. Luzarowski M. Kerber O. Leniak E. Skirycz A. (2018). Interaction of 2’,3’-cAMP with Rbp47b plays a role in stress granule formation. *Plant Physiol.* 177 00285.02018. 10.1104/pp.18.00285 29618637 PMC5933139

[B81] Kuhn J. M. (2003). Impacts of altered RNA metabolism on abscisic acid signaling. *Curr. Opin. Plant Biol.* 6 463–469. 10.1016/S1369-5266(03)00084-0 12972047

[B82] Kwak K. J. Kim H.-S. Jang H. Y. Kang H. Ahn S.-J. (2016). Diverse roles of glycine-rich RNA-binding protein 7 in the response of camelina (*Camelina sativa*) to abiotic stress. *Acta Physiol. Plant.* 38:129. 10.1007/s11738-016-2144-4

[B83] Kwak K. J. Kim Y. O. Kang H. (2005). Characterization of transgenic *Arabidopsis* plants overexpressing GR-RBP4 under high salinity, dehydration, or cold stress. *J. Exp. Bot.* 56 3007–3016. 10.1093/jxb/eri298 16207746

[B84] Kwak K. J. Park S. J. Han J. H. Kim M. K. Oh S. H. Han Y. S. (2011). Structural determinants crucial to the RNA chaperone activity of glycine-rich RNA-binding proteins 4 and 7 in *Arabidopsis thaliana* during the cold adaptation process. *J. Exp. Bot.* 62 4003–4011. 10.1093/jxb/err101 21511907 PMC3134357

[B85] Kwanuk L. Jung P. S. Youn-Il P. Kang H. (2019). CFM9, a mitochondrial CRM protein, is crucial for mitochondrial intron splicing, mitochondria function, and *Arabidopsis* growth and stress responses. *Plant Cell Physiol.* 60 2538–2548. 10.1093/pcp/pcz147 31359042

[B86] Laluk K. Abuqamar S. Mengiste T. (2011). The *Arabidopsis* mitochondria-localized pentatricopeptide repeat protein PGN functions in defense against necrotrophic fungi and abiotic stress tolerance. *Plant Physiol.* 156 2053–2068. 10.1104/pp.111.177501 21653783 PMC3149943

[B87] Lambermon M. H. Fu Y. Wieczorek Kirk D. A. Dupasquier M. Filipowicz W. Lorkovic Z. J. (2002). UBA1 and UBA2, two proteins that interact with UBP1, a multifunctional effector of pre-mRNA maturation in plants. *Mol. Cell. Biol.* 22 4346–4357. 10.1128/MCB.22.12.4346-4357.2002 12024044 PMC133861

[B88] Lee B. H. Kapoor A. Zhu J. H. Zhu J. K. (2006). STABILIZED1, a stress-upregulated nuclear protein, is required for pre-mRNA splicing, mRNA turnover, and stress tolerance in *Arabidopsis*. *Plant Cell* 18 1736–1749. 10.1105/tpc.106.042184 16751345 PMC1488911

[B89] Lee E. K. (2012). Post-translational modifications of RNA-binding proteins and their roles in RNA granules. *Curr. Protein Peptide Sci.* 13 331–336. 10.2174/138920312801619411 22708487

[B90] Lee K. Kang H. (2016). Emerging roles of RNA-binding proteins in plant growth, development, and stress responses. *Mol. Cells* 39 179–185. 10.14348/molcells.2016.2359 26831454 PMC4794599

[B91] Lee K. Kang H. (2020). Roles of organellar RNA-binding proteins in plant growth, development, and abiotic stress responses. *Int. J. Mol. Sci.* 21:4548. 10.3390/ijms21124548 32604726 PMC7352785

[B92] Lee K. Lee H. Kim D. Jeon Y. Pai H. S. Kang H. (2014). A nuclear-encoded chloroplast protein harboring a single CRM domain plays an important role in the *Arabidopsis* growth and stress response. *BMC Plant Biol.* 14:98. 10.1186/1471-2229-14-98 24739417 PMC4021458

[B93] Lee K. Park S. J. Park Y. R. Kang H. S. (2019). CFM9, a mitochondrial CRM protein, is crucial for mitochondrial intron splicing, mitochondria function, and *Arabidopsis* growth and stress responses. *Plant Cell Physiol.* 11 2538–2548. 10.1093/pcp/pcz147 31359042

[B94] Lee M. O. Kim K. P. Kim B. G. Hahn J. S. Hong C. B. (2009). Flooding stress-induced glycine-rich RNA-binding protein from *Nicotiana tabacum*. *Mol. Cells* 27 47–54. 10.1007/s10059-009-0004-4 19214433

[B95] Leene J. V. Han C. Gadeyne A. Eeckhout D. Matthijs C. Cannoot B. (2019). Capturing the phosphorylation and protein interaction landscape of the plant TOR kinase. *Nat. Plants* 5 316–327. 10.1038/s41477-019-0378-z 30833711

[B96] Li J. Kinoshita T. Pandey S. Ng K. Y. Gygi S. P. Shimazaki K. I. (2002). Modulation of an RNA-binding protein by abscisic-acid-activated protein kinase. *Nature* 418 793–797. 10.1038/nature00936 12181571

[B97] Li J. Wang X. Q. Watson M. B. Assmann S. M. (2000). Regulation of abscisic acid-induced stomatal closure and anion channels by guard cell AAPK kinase. *Curr. Opin. Plant Biol.* 3:172. 10.1016/S1369-5266(00)80031-X 10634783

[B98] Li P. Banjade S. Cheng H. C. Kim S. Chen B. Guo L. (2012). Phase transitions in the assembly of multivalent signalling proteins. *Nature* 483 336–340. 10.1038/nature10879 22398450 PMC3343696

[B99] Li Y. Guo Q. Liu P. Huang J. Zhang S. Yang G. (2021). Dual roles of the serine/arginine-rich splicing factor SR45a in promoting and interacting with nuclear cap-binding complex to modulate the salt-stress response in *Arabidopsis*. *New Phytol.* 230 641–655. 10.1111/nph.17175 33421141

[B100] Lin P. C. Pomeranz M. C. Jikumaru Y. Kang S. G. Hah C. Fujioka S. (2011). The *Arabidopsis* tandem zinc finger protein AtTZF1 affects ABA- and GA-mediated growth, stress and gene expression responses. *Plant J.* 65 253–268. 10.1111/j.1365-313X.2010.04419.x 21223390

[B101] Lin Y. Protter D. S. Rosen M. K. Parker R. (2015). Formation and maturation of phase-separated liquid droplets by RNA-binding proteins. *Mol. Cell* 60 208–219. 10.1016/j.molcel.2015.08.018 26412307 PMC4609299

[B102] Liu H. H. Liu J. Fan S. L. Song M. Z. Han X. L. Liu F. (2008). Molecular cloning and characterization of a salinity stress-induced gene encoding DEAD-box helicase from the halophyte *Apocynum venetum*. *J. Exp. Bot.* 59 633–644. 10.1093/jxb/erm355 18272921

[B103] Liu J. M. Zhao J. Y. Lu P. P. Chen M. Guo C. H. Xu Z. S. (2016). The E-subgroup pentatricopeptide repeat protein family in *Arabidopsis thaliana* and confirmation of the responsiveness PPR96 to abiotic stresses. *Front. Plant Sci.* 7:1825. 10.3389/fpls.2016.01825 27994613 PMC5136568

[B104] Liu X. Lan J. Huang Y. Cao P. Zhou C. Ren Y. (2018). WSL5, a pentatricopeptide repeat protein, is essential for chloroplast biogenesis in rice under cold stress. *J. Exp. Bot.* 69 3949–3961. 10.1093/jxb/ery214 29893948 PMC6054151

[B105] Liu Y. He J. N. Chen Z. Z. Ren X. Z. Hong X. H. Gong Z. Z. (2010). ABA overly-sensitive5 (ABO5), encoding a pentatricopeptide repeat protein required for cis-splicing of mitochondrial nad2 intron3, is involved in the abscisic acid response in *Arabidopsis*. *Plant J.* 63 749–765. 10.1111/j.1365-313X.2010.04280.x 20561255

[B106] Long R. Yang Q. Kang J. Zhang T. Wang H. Li M. (2013). Overexpression of a novel salt stress-induced glycine-rich protein gene from alfalfa causes salt and ABA sensitivity in *Arabidopsis*. *Plant Cell Rep.* 32 1289–1298. 10.1007/s00299-013-1443-0 23584549

[B107] Lorković Z. J. (2009). Role of plant RNA-binding proteins in development, stress response and genome organization. *Trends Plant Sci.* 14 229–236. 10.1016/j.tplants.2009.01.007 19285908

[B108] Lorković Z. J. Brarta A. (2002). Genome analysis: RNA recognition motif (RRM) and K homology (KH) domain RNA-binding proteins from the flowering plant *Arabidopsis thaliana*. *Nucleic Acids Res.* 30 623–635. 10.1093/nar/30.3.623 11809873 PMC100298

[B109] Lorković Z. J. Kirk D. A. W. Klahre U. Hemmings-Mieszczak M. Filipowicz W. (2000). RBP45 and RBP47, two oligouridylate-specific hnRNP-like proteins interacting with poly(A)+ RNA in nuclei of plant cells. *RNA* 6 1610–1624. 10.1017/s1355838200001163 11105760 PMC1370030

[B110] Lorsch J. R. (2002). RNA chaperones exist and DEAD box proteins get a life. *Cell* 109 797–800. 10.1016/S0092-8674(02)00804-8 12110176

[B111] Lu C. Fedoroff N. (2000). A mutation in the *Arabidopsis* HYL1 gene encoding a dsRNA binding protein affects responses to abscisic acid, auxin, and caytokinin. *Plant Cell* 12 2351–2366. 10.2307/3871234 11148283 PMC102223

[B112] Lu C. A. Huang C. K. Huang W. S. Huang X. X. Chen Y. F. (2019). DEAD-box RNA helicase 42 plays a critical role in pre-mRNA splicing under cold stress. *Plant Physiol.* 182 255–271. 10.1104/pp.19.00832 31753844 PMC6945872

[B113] Luo Y. Na Z. Slavoff S. A. (2018). P-bodies: composition, properties, and functions. *Biochemistry* 57 2424–2431. 10.1021/acs.biochem.7b01162 29381060 PMC6296482

[B114] Mahboubi H. Stochaj U. (2017). Cytoplasmic stress granules: dynamic modulators of cell signaling and disease. *Biochim. Biophys. Acta Mol. Basis Dis.* 1863 884–895. 10.1016/j.bbadis.2016.12.022 28095315

[B115] Mangeon A. Junqueira R. M. Sachetto-Martins G. (2010). Functional diversity of the plant glycine-rich proteins superfamily. *Plant Signal. Behav.* 5 99–104. 10.4161/psb.5.2.10336 20009520 PMC2884108

[B116] Maris C. Dominguez C. Allain F. H. T. (2005). The RNA recognition motif, a plastic RNA-binding platform to regulate post-transcriptional gene expression. *FEBS J.* 272 2118–2131. 10.1111/j.1742-4658.2005.04653.x 15853797

[B117] Marondedze C. (2020). The increasing diversity and complexity of the RNA-binding protein repertoire in plants. *Proc. R. Soc. B Biol. Sci.* 287:20201397. 10.1098/rspb.2020.1397 32962543 PMC7542812

[B118] Marondedze C. Thomas L. Gehring C. Lilley K. S. (2019). Changes in the *Arabidopsis* RNA-binding proteome reveal novel stress response mechanisms. *BMC Plant Biol.* 19:139. 10.1186/s12870-019-1750-x 30975080 PMC6460520

[B119] Maruri-López I. Aviles-Baltazar N. Y. Buchala A. Serrano M. (2019). Intra and extracellular journey of the phytohormone salicylic acid. *Front. Plant Sci.* 10:423. 10.3389/fpls.2019.00423 31057566 PMC6477076

[B120] Maruri-López I. Figueroa N. E. Hernandez-Sanchez I. E. Chodasiewicz M. (2021). Plant stress granules: trends and beyond. *Front. Plant Sci.* 12:722643. 10.3389/fpls.2021.722643 34434210 PMC8381727

[B121] Masliah G. Barraud P. Allain F. H. (2013). RNA recognition by double-stranded RNA binding domains: a matter of shape and sequence. *Cell. Mol. Life Sci.* 70 1875–1895. 10.1007/s00018-012-1119-x 22918483 PMC3724394

[B122] Mehrotra R. Bhalothia P. Bansal P. Basantani M. K. Bharti V. Mehrotra S. (2014). Abscisic acid and abiotic stress tolerance - different tiers of regulation. *J. Plant Physiol.* 171 486–496. 10.1016/j.jplph.2013.12.007 24655384

[B123] Miller C. L. (1900). Viruses cellular stress eIF2α phophorylation innate immunity replication stress granules translation. *Future Virol.* 6 1329–1338. 10.2217/fvl.11.108 26388931 PMC4574952

[B124] Molina A. Mena M. Carbonero P. Garcia-Olmedo F. (1997). Differential expression of pathogen-responsive genes encoding two types of glycine-rich proteins in barley. *Plant Mol. Biol.* 33 803–810. 10.1023/A:1005712803130 9106504

[B125] Molliex A. Temirov J. Lee J. Coughlin M. Kanagaraj A. P. Kim H. J. (2015). Phase separation by low complexity domains promotes stress granule assembly and drives pathological fibrillization. *Cell* 163 123–133. 10.1016/j.cell.2015.09.015 26406374 PMC5149108

[B126] Muthusamy M. Kim J.-H. Kim J. A. Lee S.-I. (2021). Plant RNA binding proteins as critical modulators in drought, high salinity, heat, and cold stress responses: an updated overview. *Int. J. Mol. Sci.* 22:6731. 10.3390/ijms22136731 34201749 PMC8269355

[B127] Muthusamy M. Yoon E. K. Kim J. A. Jeong M. J. Lee S. I. (2020). Brassica rapa SR45a regulates drought tolerance via the alternative splicing of target genes. *Genes* 11:182. 10.3390/genes11020182 32050656 PMC7074037

[B128] Nagai K. Oubridge C. Ito N. Avis J. Evans P. (1995). The RNP domain: a sequence-specific RNA-binding domain involved in processing and transport of RNA. *Trends Biochem. Sci.* 20 235–240. 10.1016/S0968-0004(00)89024-6 7543225

[B129] Nakaminami K. Matsui A. Shinozaki K. Seki M. (2012). RNA regulation in plant abiotic stress responses. *Biochim. Biophys. Acta* 1819 149–153. 10.1016/j.bbagrm.2011.07.015 21840431

[B130] Nawaz G. Kang H. (2019). Rice OsRH58, a chloroplast DEAD-box RNA helicase, improves salt or drought stress tolerance in *Arabidopsis* by affecting chloroplast translation. *BMC Plant Biol.* 19:17. 10.1186/s12870-018-1623-8 30626336 PMC6327599

[B131] Nawaz G. Lee K. Su J. P. Kim Y. O. Kang H. (2018). A chloroplast-targeted cabbage DEAD-box RNA helicase BrRH22 confers abiotic stress tolerance to transgenic *Arabidopsis* plants by affecting translation of chloroplast transcripts. *Plant Physiol. Biochem.* 127 336–342. 10.1016/j.plaphy.2018.04.007 29653436

[B132] Ng L. M. Melcher K. Teh B. T. Xu H. E. (2014). Abscisic acid perception and signaling: structural mechanisms and applications. *Acta Pharmacol. Sin.* 35 567–584. 10.1038/aps.2014.5 24786231 PMC4813750

[B133] Nguyen C. C. Nakaminami K. Matsui A. Kobayashi S. Kurihara Y. Toyooka K. (2016). Oligouridylate binding protein 1b plays an integral role in plant heat stress tolerance. *Front. Plant Sci.* 7:853. 10.3389/fpls.2016.00853 27379136 PMC4911357

[B134] Nguyen C. C. Nakaminami K. Matsui A. Watanabe S. Kanno Y. Seo M. (2017). Overexpression of oligouridylate binding protein 1b results in ABA hypersensitivity. *Plant Signal. Behav.* 12:e1282591. 10.1080/15592324.2017.1282591 28112571 PMC5351729

[B135] Nguyen L. V. Seok H. Y. Woo D. H. Lee S. Y. Moon Y. H. (2018). Overexpression of the DEAD-Box RNA helicase gene AtRH17 confers tolerance to salt stress in *Arabidopsis*. *Int. J. Mol. Sci.* 19:3777. 10.3390/ijms19123777 30486488 PMC6321491

[B136] Niewidok B. Igaev M. de Graca A. P. Strassner A. Lenzen C. Richter C. P. (2018). Single-molecule imaging reveals dynamic biphasic partition of RNA-binding proteins in stress granules. *J. Cell Biol.* 217 1303–1318. 10.1083/jcb.201709007 29463567 PMC5881506

[B137] Nomata T. Kabeya Y. Sato N. (2004). Cloning and characterization of glycine-rich RNA-binding protein cDNAs in the moss *Physcomitrella patens*. *Plant Cell Physiol.* 45 48–56. 10.1093/pcp/pch005 14749485

[B138] Ntountoumi C. Panayotis V. Dimitris M. Constantinos S. Ioannis I. Vasilios P. (2019). Low complexity regions in the proteins of prokaryotes perform important functional roles and are highly conserved. *Nucleic Acids Res.* 47 9998–10009. 10.1093/nar/gkz730 31504783 PMC6821194

[B139] Ortega-Amaro M. A. Rodríguez-Hernández A. A. Hernández-Lucero E. Jiménez-Bremont J. F. Rodríguez-Kessler M. Rosales-Mendoza S. (2014). Overexpression of AtGRDP2, a novel glycine-rich domain protein, accelerates plant growth and improves stress tolerance. *Front. Plant Sci.* 5:782. 10.3389/fpls.2014.00782 25653657 PMC4299439

[B140] Owttrim G. W. (2006). RNA helicases and abiotic stress. *Nucleic Acids Res.* 34 3220–3230. 10.1093/nar/gkl408 16790567 PMC1484253

[B141] Paieri F. Tadini L. Manavski N. Kleine T. Ferrari R. Morandini P. (2018). The DEAD-box RNA helicase RH50 is a 23S-4.5S rRNA maturation factor that functionally overlaps with the plastid signaling factor GUN1. *Plant Physiol.* 176 634–648. 10.1104/pp.17.01545 29138350 PMC5761802

[B142] Palusa S. G. Ali G. S. Reddy A. S. N. (2007). Alternative splicing of pre-mRNAs of *Arabidopsis* serine/arginine-rich proteins: regulation by hormones and stresses. *Plant J.* 49 1091–1107. 10.1111/j.1365-313X.2006.03020.x 17319848

[B143] Papp I. Mur L. A. Dalmadi A. Dulai S. Koncz C. (2004). A mutation in the Cap Binding Protein 20 gene confers drought tolerance to *Arabidopsis*. *Plant Mol. Biol.* 55 679–686. 10.1007/s11103-004-1680-2 15604709

[B144] Park H. Y. Kang I. S. Han J. S. Lee C. H. An G. Moon Y. H. (2009). OsDEG10 encoding a small RNA-binding protein is involved in abiotic stress signaling. *Biochem. Biophys. Res. Commun.* 380 597–602. 10.1016/j.bbrc.2009.01.131 19285007

[B145] Park Y. R. Choi M. J. Park S. J. Kang H. S. (2017). Three zinc-finger RNA-binding proteins in cabbage (*Brassica rapa*) play diverse roles in seed germination and plant growth under normal and abiotic stress conditions. *Physiol. Plant.* 159 93–106. 10.1111/ppl.12488 27528428

[B146] Pham J. W. Pellino J. L. Lee Y. S. Carthew R. W. Sontheimer E. J. (2004). A Dicer-2-dependent 80s complex cleaves targeted mRNAs during RNAi in *Drosophila*. *Cell* 117 83–94. 10.1016/S0092-8674(04)00258-2 15066284

[B147] Phillips R. S. Ramos S. B. V. Blackshear P. J. (2002). Members of the tristetraprolin family of tandem CCCH zinc finger proteins exhibit CRM1-dependent nucleocytoplasmic shuttling. *J. Biol. Chem.* 277:11606. 10.1074/jbc.M111457200 11796723

[B148] Pomeranz M. Lin P. C. Finer J. Jang J. C. (2010). AtTZF gene family localizes to cytoplasmic foci. *Plant Signal. Behav.* 5 190–192. 10.4161/psb.5.2.10988 20173417 PMC2884132

[B149] Pomeranz M. C. Hah C. Lin P. C. Kang S. G. Finer J. J. Blackshear P. J. (2010). The *Arabidopsis* tandem zinc finger protein AtTZF1 traffics between the nucleus and cytoplasmic foci and binds both DNA and RNA. *Plant Physiol.* 152 151–165. 10.1104/pp.109.145656 19897605 PMC2799353

[B150] Raab S. Toth Z. de Groot C. Stamminger T. Hoth S. (2006). ABA-responsive RNA-binding proteins are involved in chloroplast and stromule function in *Arabidopsis* seedlings. *Planta* 224 900–914. 10.1007/s00425-006-0282-4 16633814

[B151] Rajkowitsch L. Chen D. Stampfl S. Semrad K. Waldsich C. Mayer O. (2007). RNA chaperones, RNA annealers and RNA helicases. *RNA Biol.* 4 118–130. 10.4161/rna.4.3.5445 18347437

[B152] Reuper H. Amari K. Krenz B. (2021). Analyzing the G3BP-like gene family of *Arabidopsis thaliana* in early turnip mosaic virus infection. *Sci. Rep.* 11:2187. 10.1038/s41598-021-81276-7 33500425 PMC7838295

[B153] Riera M. Redko Y. Leung J. (2006). *Arabidopsis* RNA-binding protein UBA2a relocalizes into nuclear speckles in response to abscisic acid. *FEBS Lett.* 580 4160–4165. 10.1016/j.febslet.2006.06.064 16828085

[B154] Saad R. B. Halima N. B. Ghorbel M. Zouari N. Romdhane W. B. Guiderdoni E. (2018). AlSRG1, a novel gene encoding an RRM-type RNA-binding protein RBP from *Aeluropus littoralis*, confers salt and drought tolerance in transgenic tobacco. *Environ. Exp. Bot.* 150 25–36. 10.1016/j.envexpbot.2018.03.002

[B155] Sachetto-Martins G. Fernandes L. D. Felix D. B. de Oliveira D. E. (1995). Preferential transcriptional activity of a glycine-rich protein gene from *Arabidopsis thaliana* in protoderm-derived cells. *Int. J. Plant Sci.* 156 460–470. 10.1086/297268

[B156] Sah S. K. Reddy K. R. Li J. (2016). Abscisic acid and abiotic stress tolerance in crop plants. *Front. Plant Sci.* 7:571. 10.3389/fpls.2016.00571 27200044 PMC4855980

[B157] Sahi C. Agarwal M. Singh A. Grover A. (2007). Molecular characterization of a novel isoform of rice (*Oryza sativa* L.) glycine rich-RNA binding protein and evidence for its involvement in high temperature stress response. *Plant Sci.* 173 144–155. 10.1016/j.plantsci.2007.04.010

[B158] Sasaki K. Imai R. (2011). Pleiotropic roles of cold shock domain proteins in plants. *Front. Plant Sci.* 19:116. 10.3389/fpls.2011.00116 22639630 PMC3355641

[B159] Sasaki K. Kim M. H. Imai R. (2007). *Arabidopsis* COLD SHOCK DOMAIN PROTEIN2 is a RNA chaperone that is regulated by cold and developmental signals. *Biochem. Biophys. Res. Commun.* 364 633–638. 10.1016/j.bbrc.2007.10.059 17963727

[B160] Schmidt F. Marne F. A. Cheung M. K. Wilson I. Hancock J. Staiger D. (2010). A proteomic analysis of oligo(dT)-bound mRNP containing oxidative stress-induced *Arabidopsis thaliana* RNA-binding proteins ATGRP7 and ATGRP8. *Mol. Biol. Rep.* 37 839–845. 10.1007/s11033-009-9636-x 19672695

[B161] Sharma S. Kaur C. Singla-Pareek S. L. Sopory S. K. (2016). OsSRO1a interacts with RNA binding domain-containing protein (OsRBD1) and functions in abiotic stress tolerance in yeast. *Front. Plant Sci.* 7:62. 10.3389/fpls.2016.00062 26870074 PMC4737904

[B162] Shaw R. J. (2009). LKB1 and AMP-activated protein kinase control of mTOR signalling and growth. *Acta Physiol.* 196 65–80. 10.1111/j.1748-1716.2009.01972.x 19245654 PMC2760308

[B163] Shim J. S. Park S. H. Lee D. K. Kim Y. S. Park S. C. Redillas M. (2021). The rice GLYCINE-RICH PROTEIN 3 confers drought tolerance by regulating mRNA stability of ROS scavenging-related genes. *Rice* 14:31. 10.1186/s12284-021-00473-0 33742286 PMC7979854

[B164] Shinozuka H. Hisano H. Yoneyama S. Shimamoto Y. Jones E. S. Forster J. W. (2006). Gene expression and genetic mapping analyses of a perennial ryegrass glycine-rich RNA-binding protein gene suggest a role in cold adaptation. *Mol. Genet. Genomics* 275 399–408. 10.1007/s00438-005-0095-3 16614778

[B165] Small I. D. Peeters N. (2000). The PPR motif- a TPR-related motif prevalent in plant organellar proteins. *Trends Biochem. Sci.* 25 45–47. 10.1016/S0968-0004(99)01520-0 10664580

[B166] Small I. D. Schallenberg-Rüdinger M. Takenaka M. Mireau H. Ostersetzer-Biran O. (2020). Plant organellar RNA editing: what 30 years of research has revealed. *Plant J.* 101 1040–1056. 10.1111/tpj.14578 31630458

[B167] Sorenson R. Bailey-Serres J. (2014). Selective mRNA sequestration by OLIGOURIDYLATE-BINDING PROTEIN 1 contributes to translational control during hypoxia in *Arabidopsis*. *Proc. Natl. Acad. Sci. U.S.A.* 111 2373–2378. 10.1073/pnas.1314851111 24469793 PMC3926019

[B168] Streitner C. Hennig L. Korneli C. Staiger D. (2010). Global transcript profiling of transgenic plants constitutively overexpressing the RNA-binding protein AtGRP7. *BMC Plant Biol.* 10:221. 10.1186/1471-2229-10-221 20946635 PMC3017831

[B169] Su H. G. Li B. Song X. Y. Ma J. Chen J. Zhou Y. B. (2019). Genome-wide analysis of the DYW subgroup PPR gene family and identification of gmPPR4 responses to drought stress. *Int. J. Mol. Sci.* 20:5667. 10.3390/ijms20225667 31726763 PMC6888332

[B170] Subramanian A. R. (1983). Structure and functions of ribosomal protein S1. *Prog. Nucleic Acid Res. Mol. Biol.* 28 101–142. 10.1007/BF02754319 6348874

[B171] Tam P. P. Barrette-Ng I. H. Simon D. M. Tam M. W. Ang A. L. Muench D. G. (2010). The Puf family of RNA-binding proteins in plants: phylogeny, structural modeling, activity and subcellular localization. *BMC Plant Biol.* 10:44. 10.1186/1471-2229-10-44 20214804 PMC2848763

[B172] Tan J. J. Tan Z. H. Wu F. Q. Sheng P. Heng Y. Q. Wang X. H. (2014). A novel chloroplast-localized pentatricopeptide repeat protein involved in splicing affects chloroplast development and abiotic stress response in rice. *Mol. Plant* 7 1329–1349. 10.1093/mp/ssu054 24821718

[B173] Tanner N. K. Linder P. (2001). DExD/H Box RNA helicases. *Mol. Cell* 8 251–262. 10.1016/S1097-2765(01)00329-X 11545728

[B174] Tourriere H. Gallouzi I. E. Chebli K. Capony J. P. Mouaikel J. van der Geer P. (2001). RasGAP-associated endoribonuclease G3BP: selective RNA degradation and phosphorylation-dependent localization. *Mol. Cell. Biol.* 21 7747–7760. 10.1128/mcb.21.22.7747-7760.2001 11604510 PMC99945

[B175] Wachter A. Rühl C. Stauffer E. (2012). The role of polypyrimidine tract-binding proteins and other hnRNP proteins in plant splicing regulation. *Front. Plant Sci.* 3:81. 10.3389/fpls.2012.00081 22639666 PMC3355609

[B176] Wang B. Wang G. Shen F. Zhu S. (2018). A glycine-rich RNA-binding protein, CsGR-RBP3, is involved in defense responses against cold stress in harvested cucumber (*Cucumis sativus* L.) fruit. *Front. Plant Sci.* 9:540. 10.3389/fpls.2018.00540 29740470 PMC5925850

[B177] Wang C. Zhang D. W. Wang Y. C. Zheng L. Yang C. P. (2012). A glycine-rich RNA-binding protein can mediate physiological responses in transgenic plants under salt stress. *Mol. Biol. Rep.* 39 1047–1053. 10.1007/s11033-011-0830-2 21573794

[B178] Wang C. L. Washida H. Crofts A. J. Hamada S. Katsube-Tanaka T. Kim D. (2008). The cytoplasmic-localized, cytoskeletal-associated RNA binding protein OsTudor-SN: evidence for an essential role in storage protein RNA transport and localization. *Plant J.* 55 443–454. 10.1111/j.1365-313X.2008.03516.x 18410482

[B179] Wang X. M. Kong R. R. Zhang T. Gao Y. Y. Dong Y. J. (2020). A DEAD-box RNA helicase TCD33 that confers chloroplast development in rice at seedling stage under cold stress. *J. Plant Physiol.* 248:153138. 10.1016/j.jplph.2020.153138 32213379

[B180] Weber C. Nover L. Fauth M. (2008). Plant stress granules and mRNA processing bodies are distinct from heat stress granules. *Plant J.* 56 517–530. 10.1111/j.1365-313X.2008.03623.x 18643965

[B181] Wheeler J. R. Matheny T. Jain S. Abrisch R. Parker R. (2016). Distinct stages in stress granule assembly and disassembly. *Elife* 5:e18413. 10.7554/eLife.18413 27602576 PMC5014549

[B182] Wu L. Wu J. Liu Y. Gong X. Xu J. Lin D. (2016). The rice pentatricopeptide repeat gene TCD10 is needed for chloroplast development under cold stress. *Rice* 9:67. 10.1186/s12284-016-0134-1 27910002 PMC5133210

[B183] Xiong L. M. Gong Z. Z. Rock C. D. Subramanian S. Guo Y. Xu W. Y. (2001). Modulation of abscisic acid signal transduction and biosynthesis by an Sm-like protein in *Arabidopsis*. *Dev. Cell* 1 771–781. 10.1016/S1534-5807(01)00087-9 11740939

[B184] Xu J. Chen Y. Qian L. Mu R. Yuan X. Fang H. (2017). A novel RNA-binding protein involves ABA signaling by post-transcriptionally repressing ABI2. *Front. Plant Sci.* 8:24. 10.3389/fpls.2017.00024 28174577 PMC5258706

[B185] Xu T. Gu L. Ji C. M. Jin K. R. Chung S. M. Kang H. (2014). Comparative functional analysis of wheat (*Triticum aestivum*) zinc finger-containing glycine-rich RNA-binding proteins in response to abiotic stresses. *PLoS One* 9:e96877. 10.1371/journal.pone.0096877 24800811 PMC4011930

[B186] Yan C. Yan Z. Wang Y. Yan X. Han Y. (2014). Tudor-SN, a component of stress granules, regulates growth under salt stress by modulating GA20ox3 mRNA levels in *Arabidopsis*. *J. Exp. Bot.* 65 5933–5944. 10.1093/jxb/eru334 25205572 PMC4203129

[B187] Yang L. Zhang J. He J. Qin Y. Hua D. Duan Y. (2014). ABA-mediated ROS in mitochondria regulate root meristem activity by controlling plethora expression in *Arabidopsis*. *PLoS Genet.* 10:e1004791. 10.1371/journal.pgen.1004791 25522358 PMC4270459

[B188] Yang W. Chendrimada T. P. Wang Q. Higuchi M. Seeburg P. H. Shiekhattar R. (2006). Modulation of microRNA processing and expression through RNA editing by ADAR deaminases. *Nat. Struct. Mol. Biol.* 13 13–21. 10.1038/nsmb1041 16369484 PMC2950615

[B189] Zhang H. Zhao Y. Zhu J. K. (2020). Thriving under stress: how plants balance growth and the stress response. *Dev. Cell* 55 529–543. 10.1016/j.devcel.2020.10.012 33290694

[B190] Zhang J. Yuan H. Yang Y. Fish T. Lyi S. M. Thannhauser T. W. (2016). Plastid ribosomal protein S5 is involved in photosynthesis, plant development, and cold stress tolerance in *Arabidopsis*. *J. Exp. Bot.* 67 2731–2744. 10.1093/jxb/erw106 27006483 PMC4861020

[B191] Zhu M. Chen G. Dong T. Wang L. Zhang J. Zhao Z. (2015). SlDEAD31, a putative DEAD-Box RNA helicase gene, regulates salt and drought tolerance and stress-related genes in tomato. *PLoS One* 10:e0133849. 10.1371/journal.pone.0133849 26241658 PMC4524616

[B192] Zhu S. B. Gu J. G. Yao J. J. Li Y. C. Zhang Z. T. Xia W. C. (2022). Liquid-liquid phase separation of RBGD2/4 is required for heat stress resistance in *Arabidopsis*. *Dev. Cell* 57 583–597.e6. 10.1016/j.devcel.2022.02.005 35231447

[B193] Zimmermann P. Hirsch-Hoffmann M. Hennig L. Gruissem W. (2004). GENEVESTIGATOR. *Arabidopsis* microarray database and analysis toolbox. *Plant Physiol.* 136 2621–2632. 10.1104/pp.104.046367 15375207 PMC523327

[B194] Zsigmond L. Rigo G. Szarka A. Szekely G. Otvos K. Darula Z. (2008). *Arabidopsis* PPR40 connects abiotic stress responses to mitochondrial electron transport. *Plant Physiol.* 146 1721–1737. 10.1104/pp.107.111260 18305213 PMC2287346

